# A Mutation Losing an RBP‐Binding Site in the LncRNA NORSF Transcript Influences Granulosa Cell Apoptosis and Sow Fertility

**DOI:** 10.1002/advs.202404747

**Published:** 2024-08-09

**Authors:** Miaomiao Wang, Wenmin Sheng, Jiyu Zhang, Qiuyu Cao, Xing Du, Qifa Li

**Affiliations:** ^1^ College of Animal Science and Technology Nanjing Agricultural University Nanjing 210095 China

**Keywords:** Caspase8, NORSF, NR2C1, RNA:dsDNA triplex, sow fertility traits

## Abstract

Sow fertility is an economically important quantitative trait. Hundreds of quantitative trait loci (QTLs) containing tens of thousands of potential candidate genes are excavated. However, among these genes, non‐coding RNAs including long non‐coding RNAs (lncRNAs) are often overlooked. Here, it is reported that *NORSF* is a novel causal lncRNA for sow fertility traits in QTLs. QTLs are characterized for sow fertility traits at the genome‐wide level and identified 4,630 potential candidate lncRNAs, with 13 differentially expressed during sow follicular atresia. *NORSF*, a lncRNA that involved in sow granulosa cell (sGC) function, is identified as a candidate gene for sow fertility traits as a G to A transversion at 128 nt in its transcript is shown to be markedly associated with sow fertility traits. Mechanistically, after forming the RNA:dsDNA triplexes with the promoter of *Caspase8*, *NORSF* transcript with allele G binds to an RNA‐binding protein (RBP) NR2C1 and recruits it to the promoter of *Caspase8*, to induce *Caspase8* transcription in sGCs. Functionally, this leads to a loss of inducing effect of *NORSF* on sGC apoptosis by inactivating the death receptor‐mediated apoptotic pathway. This study identified a novel causal lncRNA that can be used for the genetic improvement of sow fertility traits.

## Graphical abstract

In sows with the genotype GG at g.128G>A of *NORSF*, after forming the RNA:dsDNA triplexes with *Caspase8* promoter, *NORSF* binds to NR2C1 and recruits it to *Caspase8* promoter, to activate *Caspase8* transcription in sGCs, trigger the death receptor‐mediated apoptotic pathway, and ultimately lead to a decrease in fertility. In sows with the genotype AA, NORSF does not affect sGC function.

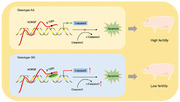

## 1 Introduction

Most biological traits such as growth traits, physiological traits, and yield traits are complex and quantitative traits. Their phenotypic variation is the result of gene‐environment interactions (GEI) and is genetically controlled by quantitative trait loci (QTLs) that harbour multiple genes, each with a small effect. Therefore, exploring specific QTLs that control quantitative traits and identifying causal variants (e.g., single nucleotide variants (SNVs) and structural variations) that explain phenotypic variations are of great significance for understanding the contribution of genetics to complex traits.^[^ ^1^ ^]^ In recent years, various high‐throughput technologies (e.g., whole genome sequencing (WGS)) have been widely applied to identify a large number of QTLs for economically important traits in various farm animals, laying the foundation for further improving production performance and benefiting humanity through molecular breeding methods.^[^ ^2^, ^3^, ^4^, ^5^, ^6^ ^]^ For example, by the end of 2022, 28961 QTLs for economically important traits have been identified, including 900 QTLs for growth traits, 4653 QTLs for meat quality traits, 1028 QTLs for disease resistance traits, and 1176 QTLs for fertility traits in pigs (https://www.animalgenome.org/cgi‐bin/QTLdb/SS/index). Because a QTL usually contains multiple (or even thousands) potential candidate genes, the vast majority of candidate genes within QTLs have not been validated, making it impossible to clearly associate QTLs with a single or few causal genes and variants. Thus, causal genes or variants that have been confirmed to control economically important traits in farm animals differ significantly from the number of potential candidate genes within these QTLs.^[^ ^6^ ^]^ In addition, a problem occurs that when analysing candidate genes within QTLs, only protein‐coding genes are often selected, whereas non‐coding RNAs (ncRNAs) (are functional untranslated RNAs that account for more than 80% of the total transcripts^[^ ^7^, ^8^ ^]^ are ignored, with the number of potential candidate genes in QTLs without protein‐coding genes is directly recorded as zero. Although some QTLs contain a large number of ncRNAs, such as microRNAs (miRNAs), long ncRNAs (lncRNAs), and circular RNAs, and some ncRNAs have been shown to regulate important economic trait‐related cellular functions and biological processes.^[^ ^9^, ^10^, ^11^ ^]^ A few ncRNAs in QTLs have been identified as causal genes affecting economically important traits, such as lncRNA MEG3 for meat production traits,^[^ ^12^ ^]^ miR‐23a for sow fertility traits.^[^ ^13^ ^]^ Therefore, the validation of candidate genes and the identification of ncRNAs within QTLs remain important directions for the genetic analysis of economically important traits and molecular breeding research in farm animals.

Sow fertility is one of the most important traits in swine production as it directly determines the overall productivity and economic benefits of the swine industry. Furthermore, sow fertility traits are the three main economic traits in swine production, along with growth traits (e.g., average daily gain [ADG]) and feed conversion ratio (FCR) traits. These three types of traits are also the main target traits for genetic improvement in swine worldwide and have made substantial genetic progress in recent decades. However, compared to the other two types of traits, the heritability of sow fertility traits is generally lower, such as 0.45 and 0.39 for the ADG and FCR traits, respectively,^[^ ^14^ ^]^ whereas less than 0.10 for the total number of piglets born alive (NBA) trait.^[^ ^15^ ^]^ Therefore, the improvement of sow fertility traits is more suitable for molecular breeding techniques based on the genetic markers of candidate genes. This also means that more candidate genes, especially causal genes, must be identified. Although nearly 1000 QTLs for sow fertility traits have been excavated and hundreds of candidate genes have been identified,^[^ ^6^, ^16^, ^17^ ^]^ this is far from meeting the needs of molecular breeding. Notably, over 4500 potential candidate lncRNAs occur within these QTLs; however, none have been confirmed as candidate lncRNAs for sow fertility traits. In this study, we provide a panoramic view of QTLs and potential candidate lncRNAs for sow fertility traits and identify a causal mutation and lncRNA for sow fertility traits.

## 2 Results

### 2.1 Genome‐Wide Identification of the Potential Candidate LncRNAs for Sow Fertility Traits in Follicles

**Figure 1:**
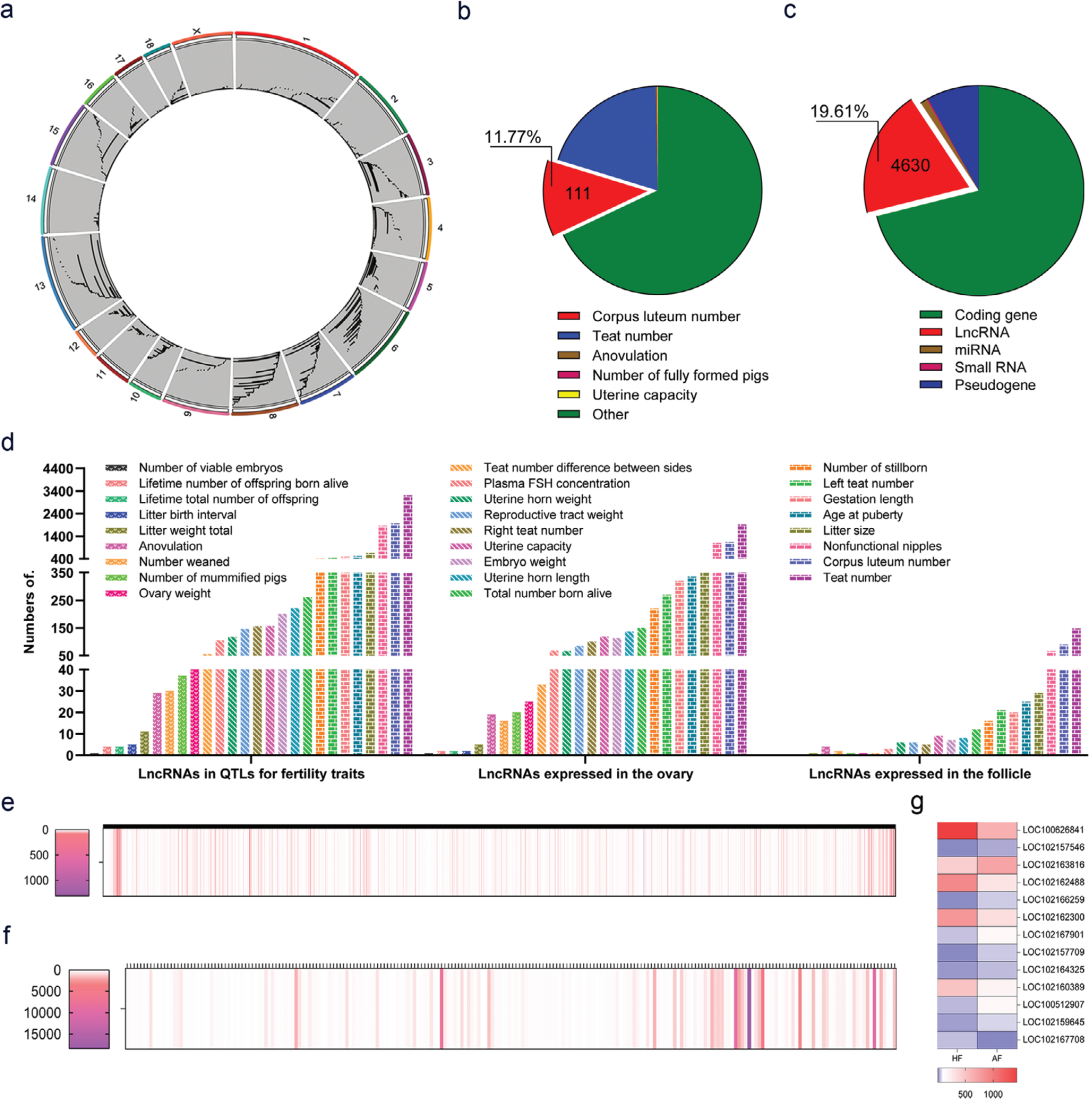
Identification of the potential candidate lncRNAs for sow fertility traits. a) Chromosomal distribution of QTLs for sow fertility traits. Data were downloaded from the pigQTLdb (https://www.animal‐genome.org/cgi‐bin/QTLdb/SS/index). b) The proportion of QTLs of various sow fertility traits. c) The proportion of various transcripts in QTLs for sow fertility traits. Genes located entirely within the QTLs were identified as potential candidate genes. d) The potential candidate lncRNAs in QTLs for sow fertility traits, expressed in sow ovaries and follicles. e,f) Heatmap of the potential candidate lncRNAs expressed in sow ovaries (e) and follicles (f). Ovarian expression data were downloaded from the NCBI database (https://www.ncbi.nlm.nih.gov/), and follicular expression data were obtained from our previous study. The scale bar stands for Reads Per Kilobase per Million (RPKM) (e) and read number (f), respectively. g) Heatmap of the potential candidate lncRNAs that differentially expressed during sow follicular atresia. RNA‐seq data were obtained from our previous study.^[^ ^11^ ^]^ The scale bar stands for read number.

A total of 943 QTLs for 28 sow fertility traits were included in the pigQTLdb database and were distributed across 19 chromosomes, including 18 autosomes and the X chromosome of pigs (**Figure** **1**a; and Table S6, Supporting Information). However, the chromosome distribution of these QTLs is uneven, with the most QTLs (8.37%, 79/943) on chromosome 1 and the least QTLs (0.95%, 9/943) on Chromosome X. Furthermore, among the 28 sow fertility traits, the number of QTLs for the teat number trait is the largest (19.94%, 188/943), followed by the corpus luteum number (ovulation rate) trait (11.77%, 111/943), and the number of QTLs for the anovulation, number of fully formed pigs and uterine capacity traits are the smallest (0.11%, 1/943) (Figure 1b; and Table S7, Supporting Information), indicating that the traits that identify more QTLs are mainly important traits (e.g., ovulation rate and litter size traits) and easily observed traits (e.g., teat number and litter size traits). Additionally, 23689 potential candidate genes were identified in these QTLs, with the highest number of protein‐coding genes accounting for 70.85% (16783/23689), followed by lncRNAs accounting for 19.54% (4630/23689) (Figure 1c), suggesting that lncRNAs are potential candidate genes for the most important sow fertility traits, apart from protein‐coding genes.

Next, we focused on potential candidate lncRNAs within the QTLs because of their large number but limited attention. Among the QTLs for sow fertility traits, 55.25% (521/943) of QTLs contain at least one potential candidate lncRNA, with a maximum of 389, averaging 28.4 (Table S8, Supporting Information). Interestingly, all sow fertility traits had potential candidate lncRNAs, with the highest number of potential candidate lncRNAs for the teat number trait (3206), followed by the ovulation rate trait (1960), and the lowest number of viable embryo trait (1) (Figure 1d). These data suggest that lncRNAs are potential candidate genes that are widely present in QTLs for sow fertility traits and may play a crucial role in the formation of sow fertility traits.

The ovary is the main reproductive organ that is strongly involved in the formation of the main sow fertility traits, such as ovulation rate and litter size. Therefore, we used the NCBI database and our previous RNA‐seq data^[^ ^11^ ^]^ to identify potential candidate lncRNAs (within QTLs for sow fertility traits) expressed in sow ovaries or follicles. A total of 2721 and 194 potential candidate lncRNAs were expressed in sow ovaries (Figure 1e; and Table S9, Supporting Information) or follicles (Figure 1f; and Table S10, Supporting Information), respectively. Furthermore, these lncRNAs were mainly located within QTLs for teat number, ovulation rate, and non‐functional nipple traits (Figure 1d). Notably, 13 lncRNAs were differentially expressed during sow follicular atresia (Figure 1g). We identified multiple potential candidate lncRNAs for sow fertility traits; however, none of these lncRNAs have been confirmed as candidate genes for sow fertility traits.

### 2.2 NORSF is a Candidate LncRNA Affecting Sow Fertility Traits

**Figure 2:**
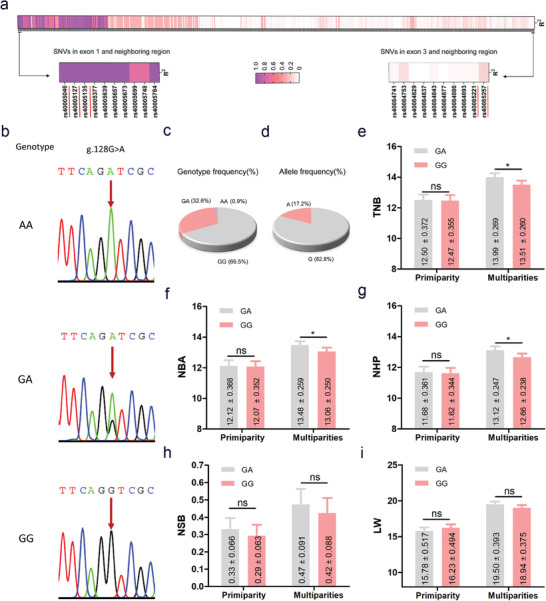
NORSF is a key candidate lncRNA affecting sow fertility traits. a) Linkage disequilibrium (LD) pattern of SNVs in *NORSF* gene. The underlines indicate SNVs in exons. Information on SNVs of *NORSF* gene was obtained from our previous low‐coverage whole‐genome data of pigs (*n* = 344). b) Sequencing peaks of different genotypes for the mutation g.128G>A. c,d) Genotype (c) and allele (d) frequency of the mutation g.128G>A in a Yorkshire sow population (*n* = 442). e–i) The fertility traits of Yorkshire sows at primiparity (*n* = 398) and multiparities (*n* = 410). e) TNB. f) NBA. g) NHP. h) NSB. i) LW. Data are plotted as the least squares mean ± standard error. *P < 0.05. ns, not significant.

Among 13 lncRNAs differentially expressed during sow follicular atresia, 5 are located in QTLs for ovarian function‐related ovulation rate trait, among which the QTL with *LOC102164325* had the strongest additive effect (Table S11, Supporting Information). In addition, *LOC102164325* (also known as *NORSF*) has also been proven to be a key regulator of sow ovarian function,^[^ ^11^ ^]^ we therefore chose *NORSF* for further analysis. To evaluate whether *NORSF* is a candidate gene for sow fertility traits, we first identified mutations in *NORSF* using our previous low‐coverage whole‐genome sequencing data.^[^ ^18^ ^]^ A total of 676 mutations were identified, of which 5 are located in exons (3 in exon1, and 2 in exon3) (**Figure** **2**a; and Table S12, Supporting Information). All five mutations in the exons are SNVs, among which the mutation rs40005046, a G to A transversion at 128 nt (also termed g.128G>A), leads to changes in the potential RNA‐binding protein (RBP)‐binding sites on the *NORSF* transcript (Figure S1 and Table S13, Supporting Information). Therefore, the mutation g.128G>A was selected as a potential causal mutation affecting sow fertility traits for further studies.

Genotyping of 442 Yorkshire sows by DNA sequencing showed that there were three genotypes of the mutation g.128G>A, and GG was the dominant genotype (294/442, 66.52%), followed by heterozygous GA (144/442, 32.58%), whereas AA was a rare genotype (4/442, <1%) (Figure 2b–d). Furthermore, the polymorphism information content of the mutation g.128G>A is 0.24 (Table S14, Supporting Information), indicating that it was low polymorphic in this sow population. Additionally, the chi‐square test showed that the mutation g.128G>A did not fit the Hardy‐Weinberg equilibrium in this sow population (*P *< 0.01).

Importantly, we showed that the mutation g.128G>A in *NORSF* was remarkably associated with sow fertility traits for multiparity, such as total number of piglets born (TNB), NBA, number of healthy piglets (NHP) (Figure 2e,i). For the TNB trait, sows with the homozygous GG were 0.48 per litter fewer than that of sows with the heterozygous GA, with a significant difference (*P* < 0.05) (Figure 2e). Additionally, the TNB of sows with the homozygous AA were 0.72 per litter higher than that of sows with the homozygous GG for multiparities, however, because there were fewer sows (*n* = 4) with the homozygous AA, no significant test was conducted. Taken together, our data suggest that *NORSF* is a novel candidate gene (lncRNA) in QTL for sow fertility traits. Additionally, the favorable genotype AA for sow fertility is the dominant genotype (76.79%, 182/237) in Erhualian pigs, a Chinese indigenous pig breed known for its high fertility, while GG is a rare genotype (1.69%, 4/237) (Figure S2, Supporting Information), with the distribution of genotypes is opposite to that in Yorkshire sows, further indicating that the mutation g.128G>A may be involved in sow prolificacy.

### 2.3 g.128G>A Mutation Causes NORSF to Lose its Ability to Induce sGC Apoptosis

**Figure 3:**
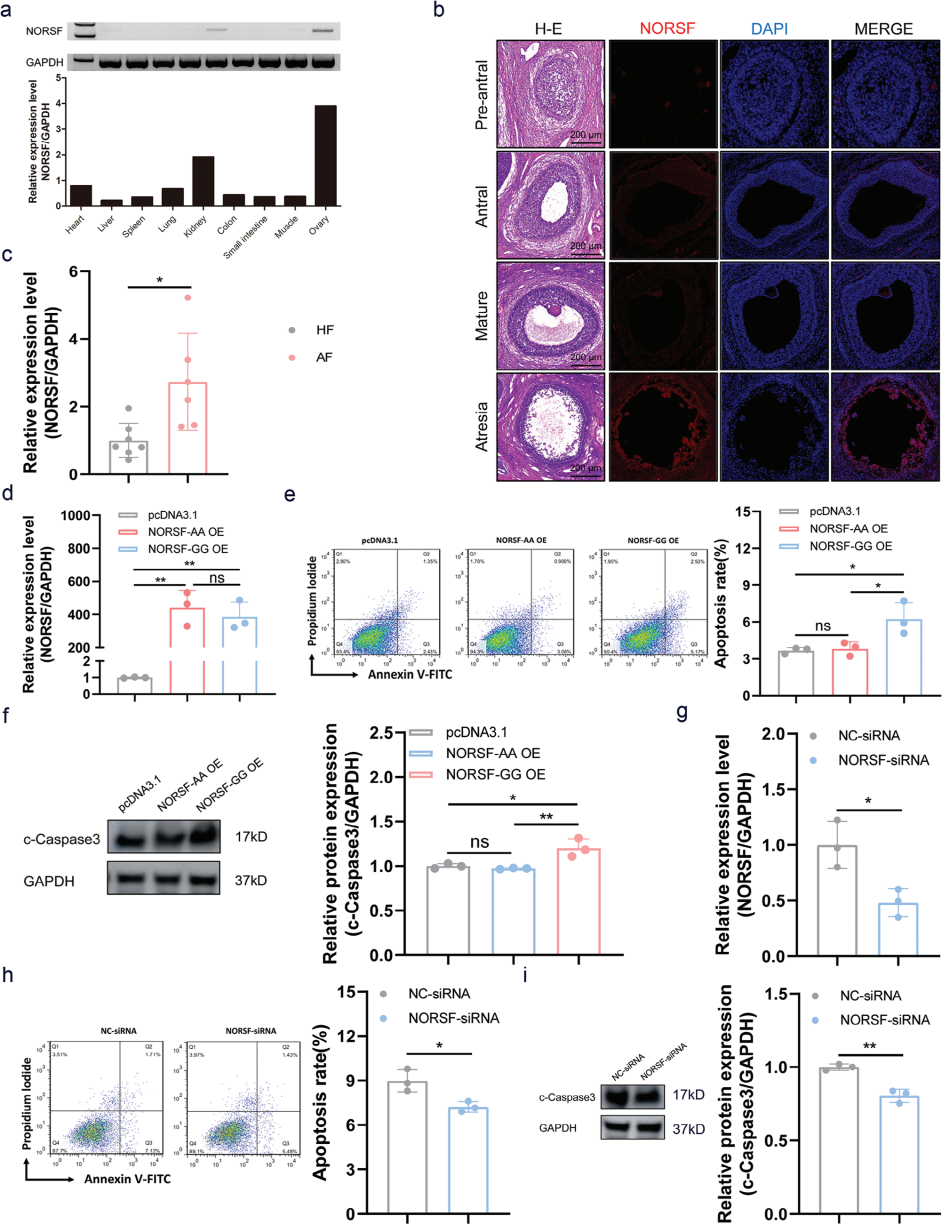
g.128G>A mutation causes NORSF to lose its ability to induce sGC apoptosis. a) Tissue expression patterns of the *NORSF* transcript in sows. b) Ovarian expression patterns of the *NORSF* transcript. Column 1 shows follicles stained with H‐E, and columns 2–4 show follicles stained with FISH. The *NORSF* transcript was shown as red, and nuclear were stained with DAPI (blue). c) The levels of *NORSF* transcript in sGCs of healthy follicles (HF) and atretic follicles (AF). HF, *n* = 7. AF, *n* = 6. d–f) sGCs were transfected with overexpression vectors of NORSF transcript with genotype GG or AA, *NORSF* levels were measured by qPCR (d), apoptosis rate was determined by FACS (e), and c‐Caspase3 levels were determined by western blotting (f). *n* = 3. g–i) sGCs were transfected with NORSF‐siRNA, *NORSF* levels were measured by qPCR (g), apoptosis rate was determined by FACS (h), and c‐Caspase3 levels were determined by western blotting (i). *n* = 3. Quantitative data are plotted as mean ± standard error. *P < 0.05. **P < 0.01. ns, not significant.

In sows, the *NORSF* transcript was predominantly expressed in the ovary, whereas its abundance was particularly low in other tissues, such as the kidney and heart (**Figure** **3**a). In the ovary, the *NORSF* transcript was lowly expressed in healthy follicles from pre‐antral follicles to mature follicles, but was highly expressed in atretic follicles, mainly stained in sGCs (Figure 3b). Furthermore, qPCR confirmed that the *NORSF* transcript levels were higher in atretic follicles than in healthy follicles (Figure 3c). These results suggest that *NORSF* is a lncRNA with abundantly expressed in the ovary and is associated with sow follicular atresia.

Sow follicular atresia is triggered by sGC apoptosis.^[^ ^9^ ^]^ To investigate the function of *NORSF* in sow follicular atresia, flow cytometry (FACS) was employed to determine the rate of apoptosis in *NORSF*‐overexpressing or ‐deficient sGCs. Ectopic expression of the *NORSF* transcript caused a marked increase in the proportion of apoptotic sGCs (Figure 3d,e). Similarly, levels of cleaved Caspase3 (c‐Caspase3), a crucial index reflecting cell apoptosis, were markedly increased in sGCs that overexpressed *NORSF* (Figure 3f). On the contrary, depleted the *NORSF* transcript leads to a remarkable decrease in the proportion of apoptotic sGCs, and a remarkable decrease in the c‐Caspase3 levels (Figure 3g–i). Taken together, the gain‐ and loss‐of‐function experiments demonstrated that *NORSF* is involved in follicular atresia by inducing sGC apoptosis.

Next, we determined whether the g.128G>A mutation influenced the regulatory effect of *NORSF* on sGC apoptosis. Therefore, we constructed an overexpression vector for the *NORSF* transcript with genotype AA (mutated type) at position g.128G>A (Figure 3d). The proportion of apoptotic cells in sGCs overexpressing the *NORSF* transcript with genotype AA was not markedly different from that in the control group but was markedly lower than that in sGCs overexpressing the *NORSF* transcript with genotype GG (Figure 3e). Consistent with this, the c‐Caspase3 levels also exhibited the same trend (Figure 3f). These results revealed that the g.128G>A mutation leads to the loss of the *NORSF*’s effect on apoptosis in sGCs.

### 2.4 g.128G>A Mutation Affects *NORSF* Transcript Binding to NR2C1, an RBP and Transcription Factor

**Figure 4:**
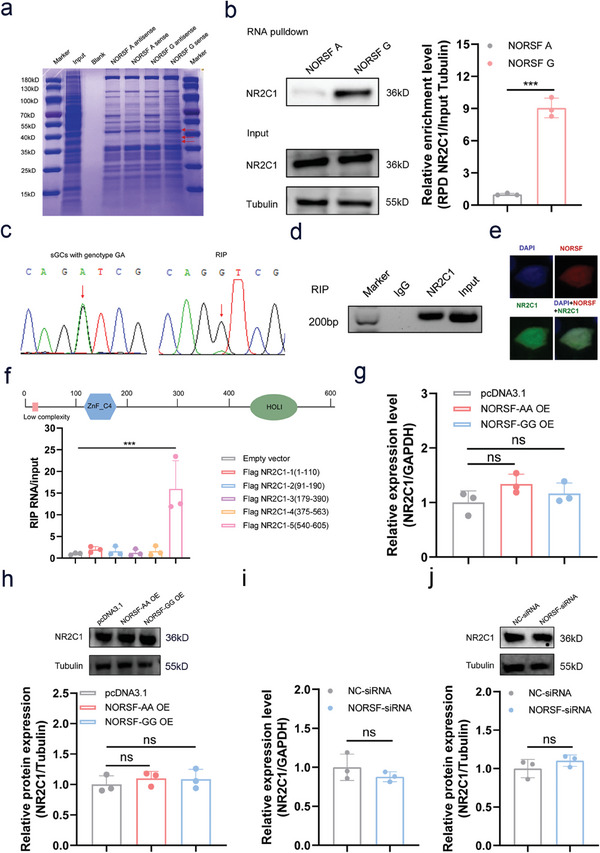
g.128G>A mutation affects NORSF transcript binding to NR2C1. a) Coomassie brilliant blue staining of RPD eluate, for which probes of *NORSF* transcript with allele G and A, and sGC lysates were used. The specific bands indicated by arrows were used for MS assay. b) Western blotting of RPD eluate to confirm the interaction of NR2C1 with *NORSF* transcript with allele G. c) Sequencing peaks of the *NORSF* transcript harboring the mutation g.128G>A in sGCs with heterozygous GA (left), and RNA products pulled down using NR2C1‐specific antibody (right). d) NR2C1 interacts with *NORSF* transcripts in sGCs genotyped as GG as detected by RIP. e) Co‐location assay of *NORSF* and NR2C1 protein in sGCs were performed by RNA‐FISH and immunofluorescence assays. Nucleus was stained with DAPI (blue), *NORSF* was dyed with NORSF‐specific probe (red), and NR2C1 protein was shown as green. f) RIP assay was conducted to detect the interaction between *NORSF* transcript and truncated fragments of NR2C1. Truncation conditioned on the intact protein structural domain of NR2C1. NR2C1‐1, NR2C1‐2, NR2C1‐3, NR2C1‐4, and NR2C1‐5 indicate different truncated fragments of NR2C1, respectively. *n* = 3. g,h) sGCs were transfected with overexpression vectors of *NORSF* transcripts with genotype GG or AA, NR2C1 mRNA (g) and protein (h) levels were measured. *n* = 3. i,j) sGCs were transfected with NORSF‐siRNA, NR2C1 mRNA (i) and protein (j) levels were measured. *n* = 3. Quantitative data are plotted as mean ± standard error. ***P < 0.001. ns, not significant.

*NORSF* is a nuclear lncRNA found in sow sGCs.^[^ ^11^ ^]^ Therefore, we speculate that *NORSF*, like other nuclear lncRNAs (e.g., *FIXER* ^[^ ^19^ ^]^ and *SUCLG2‐AS1* ^[^ ^20^ ^]^), may control the transcription of genes by recruiting transcription factors to their promoters, whereas the g.128G>A mutation may cause a decrease in the sGC apoptosis rate by altering the interaction between *NORSF* and transcription factors. To verify this hypothesis, RNA pull‐down (RPD) combined with mass spectrometry (MS) assays were conducted to identify transcription factors bound to *NORSF* transcripts with two alleles in the nuclear extract of sGCs using biotin‐labeled probes of *NORSF* transcripts with allele G or A. A total of 581 differentially bound proteins were identified, with 318 mainly interacting with the *NORSF* transcript with allele G and 263 mainly interacting with the *NORSF* transcript with allele A (**Figure** **4**a; and Table S15, Supporting Information). Furthermore, the significantly enriched gene ontology (GO) entries of the former were mainly ER to Golgi vesicle‐mediated transport (Figure S3, Supporting Information), whereas those of the latter were mainly nucleoside triphosphate metabolic process (Figure S4, Supporting Information). Interestingly, 11 of these RBPs interacting with the *NORSF* transcript with allele G are well‐known transcription factors; NR2C1 was selected for further study because it has strong binding activity with the *NORSF* transcript with the allele G (Table S15, Supporting Information), and is a transcription activator of *Caspase8*, a marker of cell apoptosis.^[^ ^21^ ^]^

RPD combined with western blot assay confirmed that the transcription factor NR2C1 interacted with the *NORSF* transcript with allele G, but not with allele A, in the nucleus of sGCs in vitro (Figure 4b). Furthermore, RNA Immunoprecipitation (RIP) experiments combined with sequencing of sGCs with heterozygous GA revealed that NR2C1 interacts with the *NORSF* transcript with allele G, but not allele A, in the nucleus of sGCs in vivo (Figure 4c). RIP assay confirmed that NR2C1 interacted with the *NORSF* transcript in sGCs with genotype GG (Figure 4d). RNA‐fluorescence in situ hybridization (RNA‐FISH) combined with immunofluorescence assays showed that *NORSF* and NR2C1 proteins were co‐located in the nuclei of sGCs (Figure 4e). Additionally, protein truncation experiments showed that the 540–605 aa region of the NR2C1 protein bound to the *NORSF* transcript in the nucleus of sGCs (Figure 4f). Together, these findings demonstrated for the first time that the transcription factor NR2C1 is an RBP that interacts with the lncRNA *NORSF* transcript with allele G for the mutation g.128G>A in sGCs.

Next, we investigated whether *NORSF* regulated NR2C1 expression in sGCs. No significant changes were observed in the mRNA and protein levels of NR2C1 in sGCs, regardless of the overexpression of the *NORSF* transcript with genotype GG or AA (Figure 4g,h), suggesting that *NORSF* does not influence NR2C1 expression in sGCs at the post‐transcriptional level, and this mutation did not affect this process. Consistent with this, there was no change in the expression of NR2C1 in sGCs that depleted the *NORSF* transcript (Figure 4i,j). Collectively, our findings revealed that the g.128G>A mutation affects the binding of *NORSF* to the RBP NR2C1 in the nuclei of sGCs.

### 2.5 NR2C1 is a Transcription Activator of *Caspase8* in sGCs

**Figure 5:**
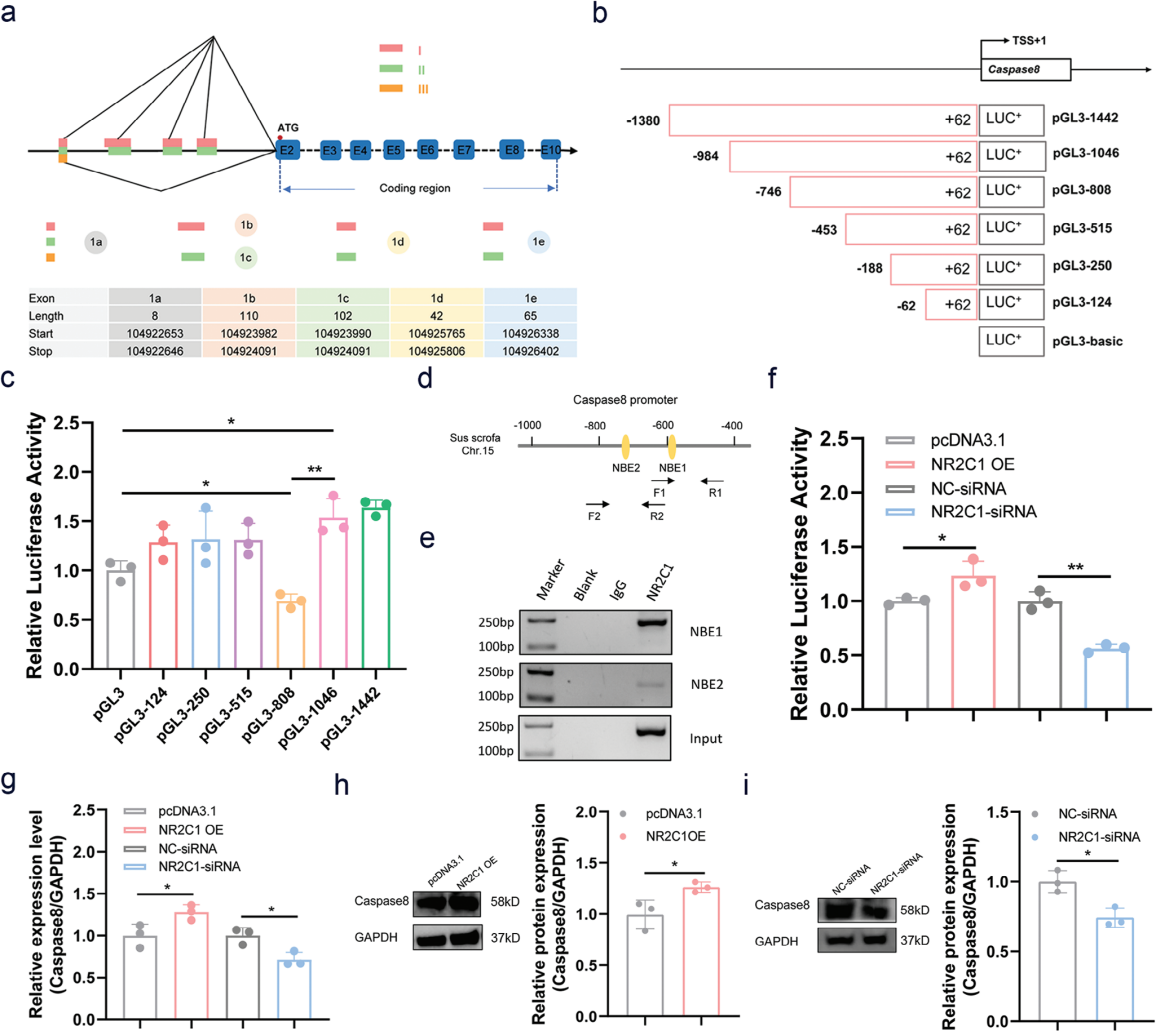
NR2C1 is a transcription activator of Caspase8 in sGCs. a) Schematic representation of *Caspase8* 5′‐UTR and transcription start site (TSS) in sGCs. *Caspase8* transcripts have a common TSS, which is located 9,256 nt before the start codon (ATG). b,c) Identification of the core promoter of the pig *Caspase8* gene in sGCs. Six deleted constructs pGL3‐124, pGL3‐250, pGL3‐515, pGL3‐808, pGL3‐1046, and pGL3‐1442 were generated (b) and transfected into KGN cells, and Luciferase activities were measured (c). *n* = 3. d,e) ChIP assays. Specific primers for the *Caspase8* promoter containing NBE motifs are designed (d) to amplify DNA products enriched by specific antibody against NR2C1 (e) in sGCs. F, forward primer. R, reverse primer. f) sGCs were co‐transfected with the reporter vector of *Caspase8* promoter and NR2C1 overexpression vector or NR2C1‐siRNA, luciferase activity was detected. *n* = 3. g) sGCs were transfected with NR2C1 overexpression vector or NR2C1‐siRNA, *Caspase8* mRNA levels were detected by qPCR. *n* = 3. h,i) sGCs were transfected with NR2C1 overexpression vector (h) or NR2C1‐siRNA (i), Caspase8 protein levels were detected by western blotting. *n* = 3. Quantitative data are plotted as mean ± standard error. *P < 0.05. **P < 0.01.

NR2C1 has been shown to be a transcription factor of *Caspase8*, which encodes an initiator of the death receptor‐mediated apoptotic pathway in mice.^[^ ^21^ ^]^ Interestingly, we also predicted that both the lncRNA *NORSF* transcript and the transcription factor NR2C1 have a strong binding ability to the porcine *Caspase8* promoter (Figure S5, Supporting Information). Therefore, we speculated that *NORSF* recruits NR2C1 to the *Caspase8* promoter to control its transcription. Here, we first verified whether NR2C1 is a transcription factor for *Caspase8* in sGCs. 5′‐rapid amplification of cDNA ends (RACE) showed that *Caspase8* has three transcripts, but their exon1 sequences were all identical, indicating that *Caspase8* transcripts has a common transcription start site (TSS) and promoter (**Figure** **5**a; Figure S6, Supporting Information). Luciferase assays of deletion constructs revealed that the DNA region from –984 nt to –747 nt is the core promoter, while the region from –746 to –453 nt is a negative regulatory region of *Caspase8* (Figure 5b,c). Two putative NR2C1‐binding elements (NBEs) were observed in these two regions (Figure 5d). Chromatin Immunoprecipitation (ChIP) assays further confirmed that NR2C1 interacted with both the NBE1 motif and the NBE2 motif within the *Caspase8* promoter in sGCs (Figure 5e). Luciferase assays revealed that *Caspase8* promoter activity was markedly increased in sGCs that overexpressed NR2C1, but markedly decreased in sGCs that depleted NR2C1 (Figure 5f; Figure S7, Supporting Information). Furthermore, ectopic expression of NR2C1 significantly increases Caspase8 levels, whereas depletion of NR2C1 significantly decreased its levels (Figure 5g–i), indicating that NR2C1 induces *Caspase8* transcription in sGCs. These results suggest that NR2C1 is a transcription activator of *Caspase8* in sGCs.

### 2.6 g.128G>A Mutation Influences *NORSF* Recruits Transcription Activator NR2C1 to *Caspase8* Promoter

**Figure 6:**
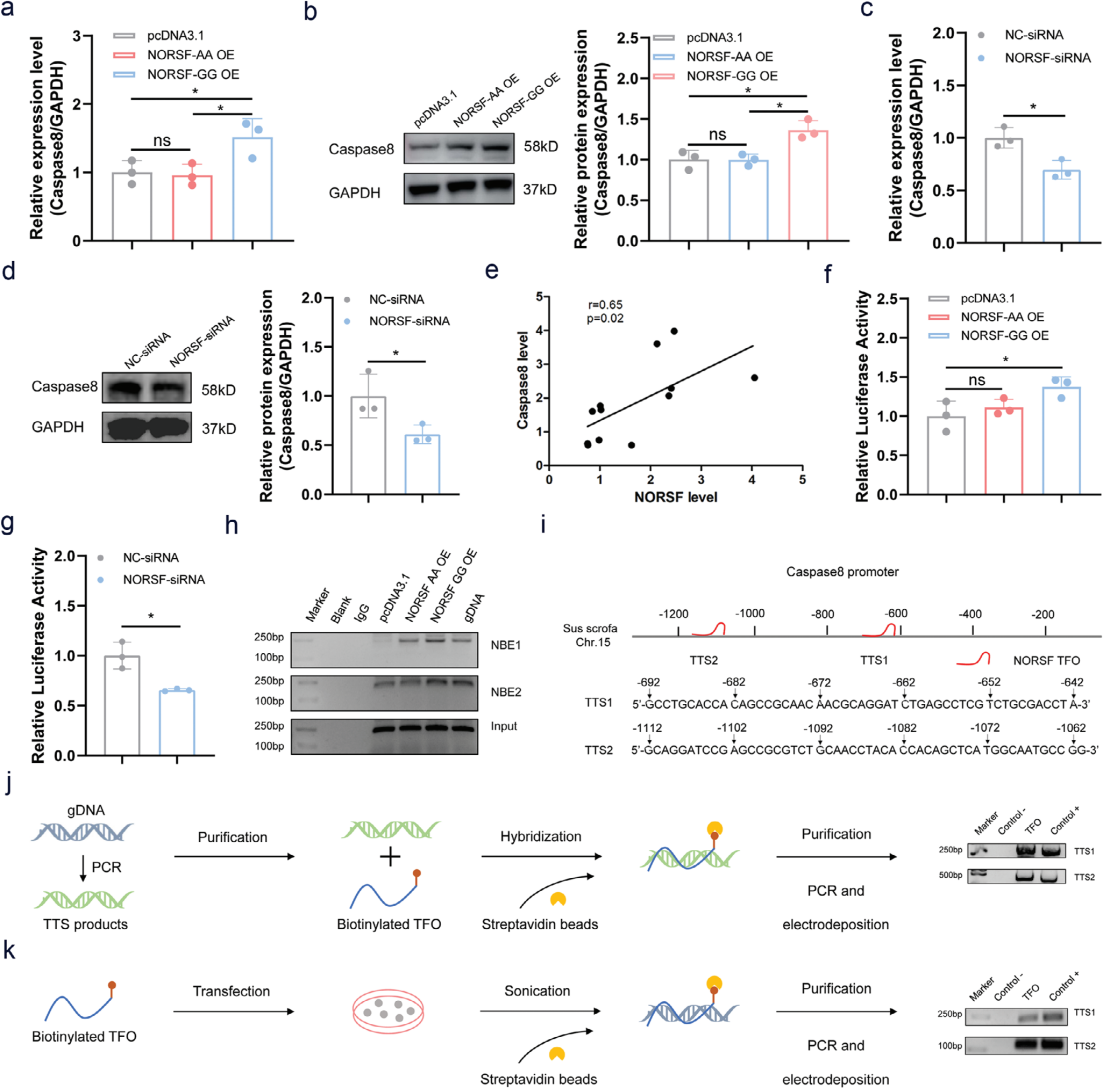
g.128G>A mutation influences NORSF recruits transcription activator NR2C1 to Caspase8 promoter. a,b) sGCs were transfected with overexpression vectors of *NORSF* transcript with genotype GG or AA, Caspase8 mRNA (a) and protein (b) levels were detected by qPCR and western blotting. *n* = 3. c,d) sGCs were transfected with NORSF‐siRNA, Caspase8 mRNA (c) and protein (d) levels were detected. *n* = 3. e) Correlation analysis between *Caspase8* levels and *NORSF* levels in sow follicles. *n* = 12. f) sGCs were co‐transfected with overexpression vectors of *NORSF* transcript with genotype GG or AA and reporter vector of *Caspase8* promoter, luciferase activity was detected. *n* = 3. g) sGCs were co‐transfected with NORSF‐siRNA and reporter vector of *Caspase8* promoter, luciferase activity was detected. *n* = 3. h) ChIP assay. Enrichment of NR2C1 in the *Caspase8* promoter in sGCs after transfection of overexpression vectors of *NORSF* transcript with genotype GG or AA was detected using an anti‐NR2C1 antibody. NBE, NR2C1‐binding element. i) Schematic showing the TTS motifs in the *Caspase8* promoter. j) In vitro triplex pull‐down assay. Schematic showing the In vitro triplex pull‐down assay by co‐transfecting with *Caspase8* promoter containing TTS motifs and biotinylated TFO probes into sGCs (left). The RNA‐interacted DNA was detected by PCR and normalized to the input DNA, and PCR products were electrophoresed in an agarose gel (right). k) In vivo triplex capture assay. Schematic showing the in vivo triplex capture assay by transfecting biotinylated TFO probes into sGCs (left). The RNA‐interacted DNA was detected by PCR and normalized to the input DNA, and PCR products were electrophoresed in an agarose gel (right). Quantitative data are plotted as mean ± standard error. *P < 0.05. ns, not significant.

Next, we investigated whether the g.128G>A mutation influences Caspase8 expression in sGCs. An increased *Caspase8* mRNA levels were observed in sGCs overexpressing the *NORSF* transcript with genotype GG, but not with genotype AA (**Figure** **6**a), indicating that the g.128G>A mutation influences the *NORSF* regulation of *Caspase8* transcription in sGCs. The levels of Caspase8 protein also show a similar trend (Figure 6b). Furthermore, decreased Caspase8 levels were observed in sGCs that depleted the *NORSF* transcript (Figure 6c,d), revealing that *NORSF* induces *Caspase8* transcription in sGCs. In addition, a marked positive correlation between *Caspase8* and *NORSF* levels was observed in the sow follicles (Figure 6e). Similar to *NORSF*, *Caspase8* is also upregulated during sow follicular atresia (Figure S8, Supporting Information). These data suggested that *NORSF* induces *Caspase8* transcription in sGCs, and the g.128G>A mutation influences the effect of *NORSF* on *Caspase8* transcription in sGCs.

To understand the mechanism underlying the effect of the g.128G>A mutation on *Caspase8* transcription in sGCs, we first determined whether it influences *Caspase8* transcription activity. An increased *Caspase8* promoter activity was observed in sGCs that overexpressed the *NORSF* transcript with genotype GG, but not with genotype AA (Figure 6f), indicating that the g.128G>A mutation influences *NORSF* regulation of *Caspase8* transcription activity in sGCs. In contrast, decreased *Caspase8* promoter activity was observed in sGCs with depleted *NORSF* transcripts (Figure 6g), revealing that *NORSF* induces *Caspase8* transcription activity in sGCs. Furthermore, ChIP assays showed that the g.128G>A mutation affected the recruitment of NR2C1 to the *Caspase8* promoter (Figure 6h). These findings reveal that the g.128G>A mutation influences *NORSF* regulation of *Caspase8* transcription in sGCs by influencing *NORSF* recruitment of the transcription activator NR2C1 to its promoter.

Nuclear lncRNAs recruit transcription factors to control target transcription, usually by forming the RNA:dsDNA triplexes with their promoters.^[^ ^22^, ^23^ ^]^ Interestingly, two putative triplex target sites (TTSs) were detected at –1112 – –1061 nt (TTS2) and –692 – –642 nt (TTS1) in the *Caspase8* promoter, and a triplex‐forming oligonucleotide (TFO) was observed at 160–207 nt of the *NORSF* transcript (Figure 6i), suggesting that the *NORSF* transcript may form the RNA:dsDNA triplexes with the *Caspase8* promoter. In vitro triplex pull‐down assays performed by co‐incubating biotinylated TFO with the DNA fragment of the *Caspase8* promoter containing TTS motifs showed that the TFO sequence from the *NORSF* transcript could specifically form triplexes with TTS motifs in the *Caspase8* promoter (Figure 6j). This was confirmed in sGCs by in vivo triplex pull‐down assays with a biotinylated TFO probe (Figure 6k). Taken together, our findings suggest that *NORSF* forms RNA:dsDNA triplexes with the *Caspase8* promoter, and the g.128G>A mutation influences *Caspase8* transcription in sGCs by altering the enrichment of the transcription activator NR2C1 on its promoter.

### 2.7 g.128G>A Mutation Influences *NORSF* Regulation of sGC Apoptosis via the Death Receptor‐Mediated Apoptotic Pathway

We demonstrated that Caspase8 is related with sow follicular atresia. To understand its function in sow follicular atresia, FACS was used to determine the apoptosis rate in Caspase8‐overexpressing or ‐depleted sGCs. The ectopic expression of Caspase8 causes in a markedly increased the proportion of apoptotic sGCs (Figure S9a,b, Supporting Information). Similarly, c‐Caspase3 levels were markedly increased in sGCs that overexpress Caspase8 (Figure S9c, Supporting Information). In contrast, depleted Caspase8 leads to a remarkable decrease in the proportion of apoptotic sGCs and a remarkable decrease in c‐Caspase3 levels (Figure S9d–f, Supporting Information). These data suggest that Caspase8 is involved in sGC apoptosis.

**Figure 7:**
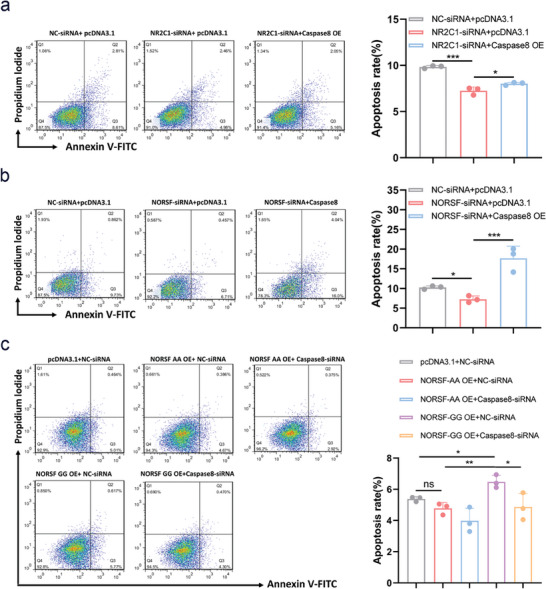
g.128G>A mutation influences NORSF regulation of sGC apoptosis via the NR2C1/Caspase8 axis. a) sGCs were co‐transfected with NR2C1‐siRNA and Caspase8 overexpression vector, and apoptosis rate was detected by FACS. *n* = 3. b) sGCs were co‐transfected with NORSF‐siRNA and Caspase8 overexpression vector, and apoptosis rate was detected by FACS. *n* = 3. c) sGCs were co‐transfected with overexpression vectors of *NORSF* transcript with genotype GG or AA, and Caspase8‐siRNA, apoptosis rate was detected by FACS. *n* = 3. Quantitative data are plotted as mean ± standard error. *P < 0.05. **P < 0.01. ***P < 0.001. ns, not significant.

NR2C1 is potentially related to sow ovarian functions, as it has the highest abundance in ovarian tissue,^[^ ^24^ ^]^ and is a transcription activator of *Caspase8*, a pro‐apoptotic factor in sGCs. Next, we investigated the role of NR2C1 in the apoptosis of sGCs. The ectopic expression of NR2C1 causes a marked raise the proportion of apoptotic sGCs (Figure S10a, Supporting Information). Similarly, c‐Caspase3 levels were markedly increased in sGCs overexpressing NR2C1 (Figure S10b, Supporting Information). In contrast, the depletion of NR2C1 led to a remarkable decrease in the proportion of apoptotic sGCs, and c‐Caspase3 levels (Figure S10c,d, Supporting Information), indicating that NR2C1 is also involved in sGC apoptosis, like Caspase8. Furthermore, rescue experiments showed that the ectopic expression of Caspase8 rescue the decrease in the proportion of apoptotic sGCs caused by NR2C1 depletion (**Figure** **7**a). Similarly, ectopic expression of Caspase8 also rescue the decrease in c‐Caspase3 levels caused by the depletion of NR2C1 (Figure S11, Supporting Information). These results revealed that NR2C1 induces sGC apoptosis by activating the death receptor‐mediated apoptotic pathway.

We also noticed that the ectopic expression of Caspase8 rescue the decrease in the proportion of apoptotic sGCs and c‐Caspase3 levels caused by *NORSF* depletion (Figure 7b; Figure S12, Supporting Information), indicating that *NORSF* induces sGC apoptosis by activating the death receptor‐mediated apoptotic pathway. To further understand whether the g.128G>A mutation influences *NORSF* regulation of sGC apoptosis through the NR2C1/Caspase8 axis, co‐transfection experiments were performed in sGCs cultured in vitro. Depleted Caspase8 represses the increase in the proportion of apoptotic sGCs caused by the *NORSF* transcript with genotype GG, while ectopic expression of the *NORSF* transcript with genotype AA had no significant effect on the sGC apoptosis rate (Figure 7c), indicating that the g.128G>A mutation influences *NORSF* regulation of sGC apoptosis through the NR2C1/Caspase8 axis. Similarly, ectopic expression of Caspase8 represses the increase in c‐Caspase3 levels caused by the *NORSF* transcript with genotype GG, whereas ectopic expression of the *NORSF* transcript with genotype AA had no significant effect on c‐Caspase3 levels (Figure S13, Supporting Information). These results suggest that the g.128G>A mutation influences *NORSF* regulation of sGC apoptosis via the death receptor‐mediated apoptotic pathway.

## 3 Discussion

LncRNAs are a class of powerful epigenetic regulators that are closely involved in various biological processes in metazoa, from the skeleton evolution of corals to neuronal cell loss in human Alzheimer's disease.^[^ ^25^, ^26^ ^]^ Similarly, hundreds of lncRNAs have also been proved to be associated with various biological processes related to sow fertility, such as follicular development and ovulation related to ovulation rate trait, and early embryo development related to the number of viable embryo trait.^[^ ^10^, ^27^ ^]^ In this study, we characterized the QTLs for sow fertility traits and identified 4630 potential candidate lncRNAs, of which 13 were related to follicular atresia, a factor that is detrimental to sow fertility. Interestingly, some lncRNAs are also *cis*‐acting lncRNAs of the candidate protein‐coding genes (even causal protein‐coding genes) for sow fertility traits in the same QTLs. *LOC100512907*, for instance, is a *cis*‐acting lncRNA of the *prolactin* (*PRL*) gene, a candidate gene for sow fertility traits, such as TNB and NBA, in a Yorkshire population,^[^ ^28^ ^]^ and both are within the same QTLs (ID: 588, 5257, and 24 284). *F‐box and leucine rich repeat protein 7* (*FBXL7*), a neighboring gene of *LOC102167708* (both are located within a QTL for sow fertility trait, ID: 7537), which has been proved to be associated with litter traits in Landrace and Yorkshire sows.^[^ ^29^ ^]^

Unfortunately, none of the thousands of potential candidate lncRNAs within the QTLs for sow fertility traits have been confirmed as candidate genes. In the present study, we identified *NORSF*, a potential candidate lncRNA in these QTLs, as a candidate gene for sow fertility traits. Thus, *NORSF* is the first lncRNA identified as a candidate gene in QTLs for sow fertility traits, providing a scientific basis for evaluating the feasibility of lncRNAs as candidate genes for sow fertility traits. *NORSF* was the first causal gene discovered within QTLs (ID: 517, 7462), which are the lncRNA‐related QTLs (lncQTLs). Taken together, our findings support the conclusion that lncRNAs, like protein‐coding genes, are potential candidate genes for economically important traits in farm animals, as they are numerous within QTLs and carry causal variants. Therefore, lncRNAs are valuable tools for exploring causal genes and variants for economically important traits and should be the focus of future research.

It is well known that low heritability traits have slow genetic progress through traditional breeding methods, as they are greatly influenced by environmental factors. In recent years, several molecular breeding strategies, such as marker‐assisted selection (MAS), genomic selection (GS), and genome editing (GE) have been gradually used to accelerate the genetic improvement of these traits in farm animals.^[^ ^30^, ^31^, ^32^ ^]^ A large number of genetic markers, especially causal variants, are required in MAS and GS because selection based on causal variants has higher accuracy.^[^ ^33^, ^34^ ^]^ GE is a revolutionary technology that has emerged in recent years and has led to biological breeding, mainly targeting the modification of one or more causal genes (especially major genes in QTLs).^[^ ^35^ ^]^ In farm animals, GE introduces favorable variants into individuals, not only providing unprecedented opportunities for effectively improving individual production performance, meat quality, and stress resistance, but also greatly shortening the breeding cycle and accelerating genetic progress.^[^ ^30^, ^36^, ^37^ ^]^ The causal variant FecB in *BMPR1B*, the first major gene for ewe fertility traits, has been successfully used as a genetic marker in the breeding of multiple new sheep strains (and breeding materials) with high fertility using MAS or GE methods; this is the best example of molecular breeding technology used in farm animal breeding.^[^ ^36^, ^38^ ^]^

At present, although a large number of potential variants related to various economically important traits have been discovered in QTLs, only some potential variants have been validated in large groups for their relationship with traits, and there are particularly few causal variants that have been confirmed to be functional and the internal mechanism of influencing target traits has been clearly annotated.^[^ ^5^, ^39^, ^40^ ^]^ This is mainly because the vast majority of potential variants are located in non‐coding regions (even “gene desert” regions), making it difficult to determine which genes are affected by these variants, making it difficult to analyze their functions and mechanisms.^[^ ^40^, ^41^ ^]^ Currently, only a dozen causal variations have been identified for sow fertility traits,^[^ ^13^, ^42^, ^43^ ^]^ which are far from meeting the requirements of molecular breeding techniques for the genetic improvement of fertility traits in sows. In this study, we identified g.128G>A in *NORSF* as a causal variant of sow fertility traits, which causes *NORSF* to lose its ability to induce sGC apoptosis and trigger follicular atresia. In summary, our findings provide a reliable genetic marker for the molecular breeding of fertility traits in sows, which needs to be further validated in future breeding practices.

Mechanistically, *NORSF* with allele G, not A, at g.128G>A, activates the transcription of the apoptotic marker Caspase8 in the nucleus of sGCs by recruiting the transcription factor NR2C1 to the Caspase8 promoter. As is well known, cytoplasmic lncRNAs mainly control the inhibition of target mRNA translation and induction of target mRNA decay via the mechanism of miRNA sponging.^[^ ^10^, ^11^ ^]^ Unlike cytoplasmic lncRNAs, nuclear lncRNAs activate or inhibit target transcription mainly by recruiting regulators, such as transcription factors and histone‐modified complexes, to target promoters or enhancers.^[^ ^19^, ^22^, ^23^, ^44^ ^]^ *SUCLG2‐AS1*, an enhancer lncRNA transcribed from the super‐enhancer of the *SOX2* gene, activates *SOX2* transcription by mediating transcription factors occupying the promoter of the latter.^[^ ^20^ ^]^ Interestingly, nuclear lncRNAs recruitment regulators control target transcription sometimes physically interact with the target promoters, that is, form an RNA:dsDNA triplex with the double‐stranded DNA in the promoter, such as, lncRNA *SUCLG2‐AS1* and *SOX2* promoter,^[^ ^20^ ^]^ lncRNA *KCNQ1OT1* and the L1 and Alu elements,^[^ ^22^ ^]^ and lncRNA *Fendrr* and the promoters of multiple targets (e.g., *TGFβ2* and *Foxf1*).^[^ ^23^ ^]^ Consistent with this, we also detected the RNA:dsDNA triplexes interacting with the *NORSF* transcript in the *Caspase8* promoter.

In our previous study, *NORSF* has been shown to control sGCs function in another way, serving as the ceRNA of miR‐339 distributed in the nucleus.^[^ ^11^ ^]^ Compared to this way, the triplex mechanism may be more prevalent in sGCs, as only 3 potential interacting miRNAs (miR‐331, miR‐339, and miR‐670) were predicted on the *NORSF* transcript, while the potential TTSs were predicted on the promoter of all 5 *cis*‐acting targets for *NORSF* (Table S17, Supporting Information). Additionally, our study demonstrates for the first time that the transcription factor NR2C1 is an RBP. With the gradual deepening of research, some classic transcription factors such as ERα and GATA4 have also been shown to serve as RBPs.^[^ ^45^, ^46^ ^]^ Further investigations are required to elucidate the role and mechanism of NR2C1 as an RBP.

As one of the two major pathways of cell apoptosis, the death receptor‐mediated apoptotic pathway is initiated by the binding of extracellular death ligands to cell surface death receptors, development of a disc‐inducing signaling complex and Caspase8 activation.^[^ ^47^ ^]^ Therefore, Caspase8 is an important target for controlling apoptosis because of its crucial role in triggering the death receptor‐mediated apoptotic pathway.^[^ ^48^, ^49^ ^]^ The transcription dysregulation or inactivation of Caspase8 influences the activity of the death receptor‐mediated apoptotic pathway and cell apoptosis.^[^ ^50^, ^51^, ^52^ ^]^ Here, we showed that the lncRNA *NORSF* and transcription factor NR2C1 axis activates the death receptor‐mediated apoptotic pathway and induces sGC apoptosis by activating Capase8 transcription. GCs are the largest group of cells within follicles and the main functional cells, and their state (proliferation or apoptosis) directly determines the fates (maturation and ovulation, or atresia and degeneration) of follicle development. Increasing evidence has demonstrated that the factors that regulate sGC apoptosis are often associated with follicular atresia, and vice versa, such as BimEL,^[^ ^53^ ^]^ miR‐423,^[^ ^54^ ^]^ and *circINHA‐001*.^[^ ^55^ ^]^ Consistent with this finding, the core components of the NORSF/Caspase8 pathway that control sGC apoptosis are strongly involved in sow follicular atresia. *NORSF*, for instance, increases during sow follicular atresia and is a positive modulator of sow follicular atresia.^[^ ^11^ ^]^ Both Caspase8 and Caspase3 are also involved in sow follicular atresia.^[^ ^56^ ^]^ Taken together, our findings reveal a new mechanism underlying the transcriptional regulation of Caspase8 and define a novel pathway that controls GC apoptosis and follicular atresia.

## 4 Conclusion

In summary, we characterized QTLs for sow fertility traits at the whole‐genome level and identified multiple potential candidate lncRNAs. Notably, *NORSF* was the first causal lncRNA in the QTLs for sow fertility traits. Mechanistically, the g.128G>A variant strongly affects sow fertility by altering the regulatory effect of *NORSF* on sGC apoptosis and follicular atresia by influencing *NORSF* recruitment of the transcription factor NR2C1 to the pro‐apoptotic *Caspase8* promoter. Overall, our findings light a new road to explore the genetic basis for the formation of sow fertility traits and provide a novel causal lncRNA and functional marker for the molecular breeding of sow fertility traits.

## 5 Experimental Section

### Bioinformatics

QTLs and tissue expression data were downloaded from the pigQTLdb (https://www.animalgenome.org/cgi‐bin/QTLdb/SS/index) and NCBI database (https://www.ncbi.nlm.nih.gov/), respectively. Data on the lncRNAome during sow follicular atresia and low‐coverage WGS were obtained from the previous studies.^[^ ^18^, ^57^ ^]^ LPLINK v1.9 software was used for LD analysis. CatRAPID (http://service.tartaglialab.com/page/catrapid_group) was used to predict RBPs potential interaction with the *NORSF* transcript. GO analysis of RBPs was conducted using Cytoscape v3.10.1 software (https://cytoscape.org/). Prediction of the potential transcription factor binding sites was conducted using JASPAR (http://jaspar.genereg.net/). Triplexator (http://bioinfor‐matics.org.au/tools/triplexator/examples.html) and IntaRNA (http://rna.informatik.uni‐freiburg.de/IntaRNA/Input.jsp ) were used for predicting RNA:dsDNA triplex, and TFO of the *NORSF* transcript and TTS in *Caspase8* promoter.

### Animals

Adult and healthy Yorkshire sows (*n* = 442) and Erhualian sows (*n* = 237) were randomly selected from Kangle (Changzhou, China) and Erhualian Seed Conservation Base (Changshu, China) for ear tissue collection. Fresh ovaries and other eight tissues (e.g., heart, liver, spleen, lung, kidney, colon, small intestine, and muscle) were collected from a slaughterhouse (Nanjing, China) to isolate sow granulosa cells (sGCs) and analyse tissue expression profiles. The experiments were approved by the Animal Ethics Committee of Nanjing Agricultural University, China.

### Genotyping and Association Analysis

DNA was extracted from ear tissues with the phenol/chloroform method. A primer pair used for genotyping the mutation g.128G>A of the NORSF transcript was as follows: 5′‐TCA AAG GGG ACA GCA CAC ATA‐3′, and 5′‐CGC CTC GGC TTC CTA CTA AAT‐3′. Association analysis was conducted using a linear model^[^ ^58^ ^]^ in SAS v9.2 software (SAS Institute, Cary, NC, USA), that included TNB, NBA, NHP, number of stillbirths (NSB), and litter weight (LW).

### RNA Isolation and Quantification

Total RNA extraction and reverse‐transcription were conducted using TRIzol reagent (Angle Gene, Nanjing, China), and a HiScriptIII First Strand cDNA Synthesis Kit (Vazyme, Nanjing, China), respectively. RNA quantification was conducted using a quantitative real‐time PCR (qPCR) with an AceQ qPCR SYBR Green Master Mix (Vazyme), and analyzed using the 2^−ΔΔCT^ method. *GAPDH* was used as a control. Primers used are listed in Table S1 (Supporting Information).

### Follicle Isolation and Classification

Follicles were isolated from fresh ovaries using a sterile scalpel, with some used to collect sGCs and others classified as healthy follicles (HFs) and atresia follicles (AFs) using previously described methods.^[^ ^55^ ^]^ ELISA assay was conducted to detect 17β‐estradiol (E2) levels using a Detection Kit (North Biotech, Beijing, China).

### FISH Assay

Fresh ovaries and sGCs were fixed with 4% paraformaldehyde and handed over to ServiceBio (Wuhan, China) for RNA‐FISH. Briefly, ovarian tissue sections were prepared and incubated with pre‐hybridization solution for 1 h at 37 °C. Probe‐containing hybridization solution was added dropwise and incubated overnight in a thermostat (Labotery, Tianjin, China). The hybridization solution was washed out, and the corresponding branching probe was added dropwise. At the end of hybridization, the hybridization solution was poured off, and the corresponding signaling probe was added dropwise. After addition of the probes, the slices were incubated with DAPI solution for 8 min, rinsed, and sealed with an anti‐fluorescence quenching sealer. Observations and photography were conducted using an Eclipse 80i microscope (Nikon, Japan). Slices stained with haematoxylin‐eosin (H‐E) served as controls. The probe sequences are 5′‐GCC TCG GCT TCC TAC TAA ATC ACC CC‐3′, and prepared by ServiceBio (Wuhan, China).

### Plasmids and Oligonucleotides

Two overexpression vectors, pcDNA3.1‐NORSF and pcDNA3.1‐NR2C1, were previously prepared.^[^ ^11^, ^13^ ^]^ The overexpression vector for *NORSF* with g.128A was synthesized by Tsingke (Nanjing, China). To construct the overexpression vector pcDNA3.1‐Caspase8, Caspase8 coding sequences were amplified from sGCs, and inserted into the pcDNA3.1 vector (Invitrogen, Carlsbad, CA, USA) between *XhoI* and *XbaI* enzyme sites. To generate luciferase reporters, *Caspase8* promoters of different lengths were amplified and inserted into the pGL3‐Basic vector (Promega, Madison, WI, USA) between *NheI* and *XhoI* enzyme sites. siRNAs targeting *NORSF*, *NR2C1*, and *Caspase8* were purchased from Generay (Shanghai, China). The primers and siRNAs used are listed in Tables S2 and S3 (Supporting Information), respectively.

### Cell Culture and Transfection

sGCs in follicles (3–5 mm) were collected with a sterile syringe and washed twice using 37 °C PBS. sGCs were inoculated into culture plates filled with DMEM/F12 medium (Gibco, Carlsbad, CA, USA) containing 15% FBS (Gibco) and 1% penicillin‐streptomycin (Gibco) and placed in a 37 °C humidified incubator with 5% CO_2_. KGN cells (human granulosa cell line) were ordered from the Cell Bank of the Chinese Academy of Science (Shanghai, China) for luciferase assay were cultured in RPMI 1640 medium (Gibco) under the above conditions. Transfection reagent was Lipofectamine 3000 (Invitrogen).

### Apoptosis Assay

After discarding the culture medium, sGCs were digested with trypsin (Thermo Fisher Scientific, Waltham, MA, USA), and washed twice with cold PBS. Next, sGCs were fixed and resuspended with 100 µL of cold binding buffer. Subsequently, 5 µL each of Annexin V‐FITC and propidium iodide (PI) (Vazyme) were added, and sGCs were stained for 10 min in the dark and resuspended with 300 µL of cold binding buffer. Apoptosis assays were performed using FACS, and analyzed using FlowJo v7.6 software (Stanford University, Stanford, USA). The equation for calculating the apoptosis rate was as follows: (sGC number in Q2 and Q3)/total sGC number.

### Western Blotting

After 48 h of transfection, western blotting was performed as previously described.^[^ ^11^ ^]^ The primary antibodies used are anti‐NR2C1 (1:1000, Abclonal, Wuhan, China), anti‐c‐Caspase3 (active) (1:1000, Sangon), anti‐Caspase8 (1:1000, Bioss), anti‐tubulin (1:2000, Proteintech, Rosemont, IL, USA), and anti‐GAPDH (1:3000, Origene, Rockville, MD, USA). GAPDH and Tubulin served as internal controls. An IgG antibody (Cell Signaling Technology, Beverly, MA, USA) was used as the secondary antibody.

### Luciferase Assay

This procedure was conducted as previously described.^[^ ^57^ ^]^ At 24 h after transfection, luciferase activity was conducted using a DLR Assay System (Promega). The relative luciferase activity was the firefly activity/renilla activity.

### RPD and MS Assays

RPD experiments were conducted as previously described.^[^ ^59^ ^]^ Briefly, biotinylated RNA and sGC lysates were rotationally incubated in a thermostat (Labotery) at 4 °C for 12 h. Streptavidin affinity beads (Merck Millipore, Schwalbach, Germany) were added and incubated at 4 °C for 1 h, and then eluted with an Elution Buffer to obtain proteins that bind to RNA. After extraction and agarose gel separation of the eluted proteins, the gel was cut and sent to Novogene (Beijing, China) for MS assay.

### RIP Assay

sGCs were collected when the cell density reached 80% and incubated overnight at 4 °C with A/G beads (Thermo Fisher Scientific) mixed with an anti‐NR2C1 antibody (Abclonal). Semiquantitative PCR was performed to detect *NORSF* transcripts in the eluted products. Primers used are listed in Table S1 (Supporting Information).

### Immunofluorescence Assay

sGCs were inoculated on cell crawls, and at the end of the incubation period, and rinsed with cold PBS, and fixed with 4% formaldehyde at 25 °C for 20 min. Incubation with anti‐NR2C1 antibody (Abclonal) and the addition of fluorescent secondary antibody (Servicebio) were performed before DAPI re‐staining of the nuclei. Images were captured using an Eclipse 80i microscope (Nikon, Tokyo, Japan).

### ChIP Assay

After adding formaldehyde to sGCs to cross‐link chromatin and proteins, NR2C1‐DNA complexes were pulled down using an anti‐NR2C1 antibody (Abclonal). Enrichment of the target DNA fragments of the *NORSF* transcript in the eluted products was determined using semi‐quantitative PCR combined with gel electrophoresis. IgG antibody (Cell Signaling Technology) was used as a negative control and untreated chromosomes were used as input controls. The primers are listed in Table S4 (Supporting Information).

### In Vitro Triplex Pull‐Down Assay

*Caspase8* promoter containing TTS motifs was amplified and purified. Approximately 150 fmol of the purified product was co‐incubated with 1 pmol of biotin‐labeled TFO probe of *NORSF* transcript in triplex hybridization solution (10 mm Tris‐HCl (PH 7.5), 20 mm KCl, 10 mm MgCl2, 0.05% Tween and 100 U of RNasin) for 2 h at 37 °C. Streptavidin‐coated affinity beads (Merck Millipore) were added and incubated for 1 h at 37 °C. The products were washed twice with wash buffer 1 (150 mm KCl, 10 mm Tris‐HCl (pH 7.5), 5 mm MgCl_2,_ 0.5% NP40, and RNasin) and once with wash buffer 2 (15 mm KCl, 10 mm Tris‐HCl (pH 7.5), and 5 mm MgCl2). RNA:dsDNA complexes were eluted with elution buffer (1% sodium dodecyl sulfate, 50 mmol L^−1^ Tris‐HCl (pH 8), and 10 mmol L^−1^ ethylene diamine tetraacetic acid (EDTA), for 5 min), and digested with RNase A (50 ng mL^−1^, 37 °C, 30 min) and proteinase K (200 ng mL^−1^, 15 °C, 15 min). After purification, the products were amplified using specific primers for Caspase8 promoter, and then electrophoresed in a 1.5% agarose gel. The PCR fragment without the *NORSF* TFO sequence was used as a negative control and products amplified using genomic DNA as a template were used as positive controls. Biotin‐tagged sequences are listed in Table S5 (Supporting Information).

### In Vivo Triplex Capture Assay

This procedure was performed as described previously.^[^ ^60^ ^]^ Briefly, 8 pmol of biotinylated TFO probe was transfected into sGCs. sGCs were collected 16 h after transfection and lysed to extract nuclear components. After digestion with 100 ng of protease K, nuclear extracts were treated with ultrasound to form ≈500 bp DNA fragments and then incubated with magnetic beads of streptavidin (Merck Millipore) in triplex hybridization buffer with shaking at 25 °C for 1 h. After elution, DNA was purified using a DNA purification kit (Tiangen, Beijing, China) and amplified using PCR. PCR products were electrophoresed on a 1.5% agarose gel. The primers are listed in Table S5 (Supporting Information).

### Statistical Analysis

Statistics were conducted using SPSS v20.0 (IBM‐SPSS, Chicago, IL, USA) and GraphPad v8.0 (San Diego, CA, USA) software. Each group contained at least three independent samples. Significance was assessed using the Student's *t*‐test with a two tailed distribution. *P* values of <0.05 was considered as statistically significant.

## Conflict of Interest

The authors declare no conflict of interest.

## Author Contributions

Q.L. and M.W. conceived and designed the research; M.W. and W.S. performed experiments; M.W., J.Z. and Q.C. analyzed the data; M.W., X.D. and Q.L. wrote the manuscript. All authors read and approved the final manuscript.

## Supporting information

Supporting Information

Supporting Information

Supplemental Tables

## Data Availability

The data that support the findings of this study are available in the supplementary material of this article.
