# Supplementary material for: A Mutation Losing an RBP‐Binding Site in the LncRNA NORSF Transcript Influences Granulosa Cell Apoptosis and Sow Fertility
Source: Adv Sci (Weinh). 2024 Aug 9;11(40):2404747. doi: 10.1002/advs.202404747 (PMC11516108; doi:10.1002/advs.202404747)
Supplement: Supplementary file 1 — Supporting Information [file ADVS-11-2404747-s003.docx]

**Supporting Information**

**A Mutation Losing an RBP-binding Site in the LncRNA NORSF Transcript Influences Granulosa Cell Apoptosis and Sow Fertility**

Miaomiao Wang, Wenmin Sheng, Jiyu Zhang, Qiuyu Cao, Xing Du, Qifa Li*

**Figure S1.**

**
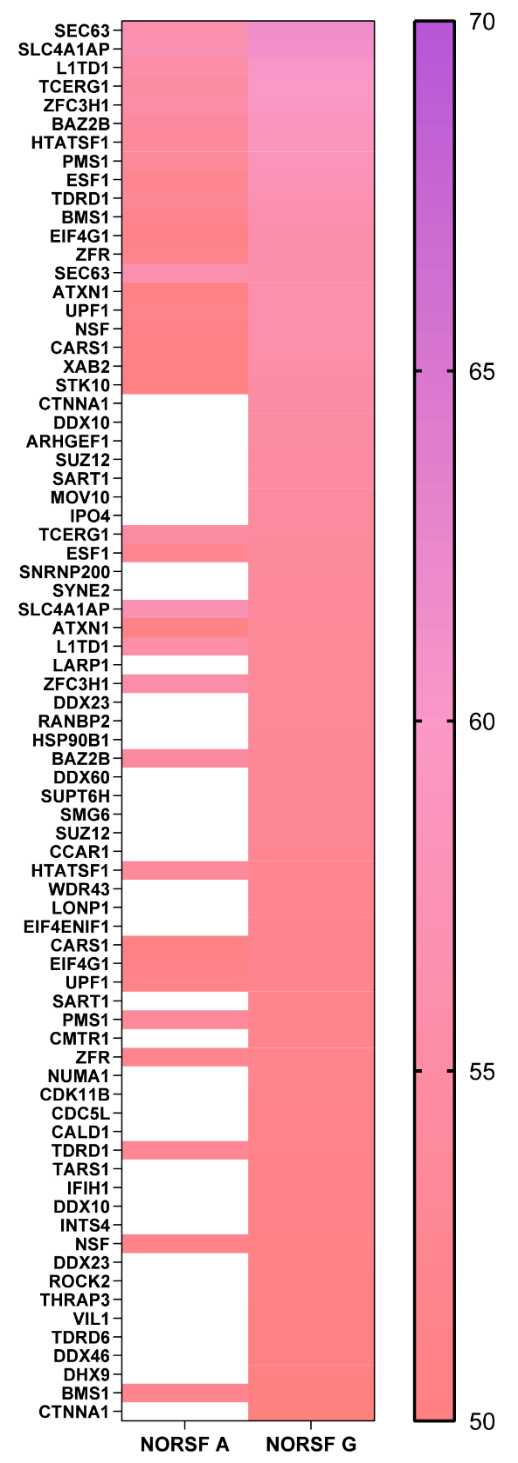
**

**Figure S1.** **RBPs potentially bound with *NORSF* transcript with allele G or A.** The potential RBPs were predicted using an online tool catRAPID (http://service. tartaglialab.com/page/catrapid_group), with the interaction propensity > 50.

**Figure S2.**

**
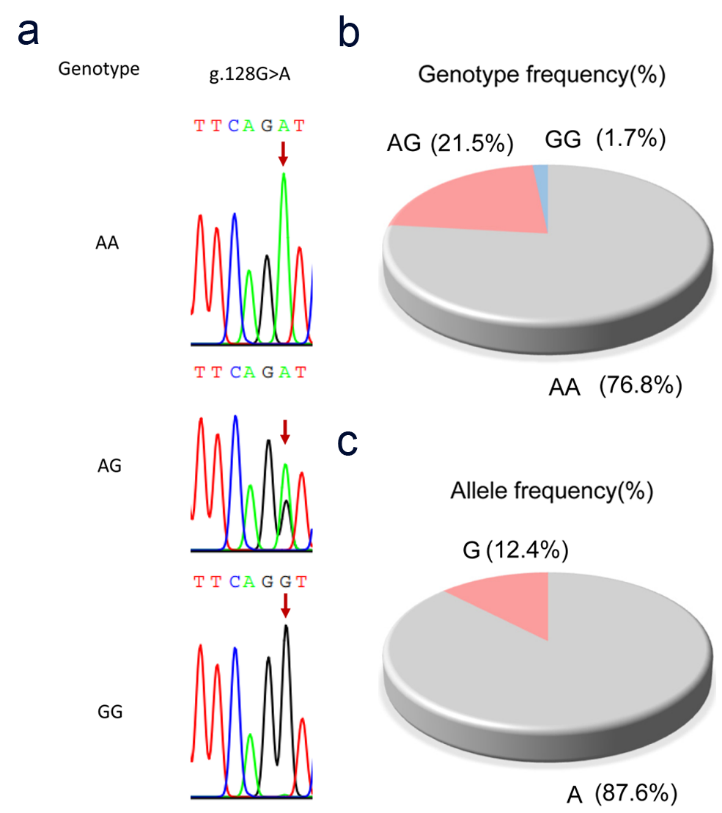
**

**Figure S2. Polymorphism of the mutation g.128G>A of *NORSF* in an Erhualian sow population.** (a) Sequencing peaks of different genotypes for the mutation g.128G>A. (b, c) Genotype (b) and allele (c) frequency of the mutation g.128G>A in an Erhualian sow population (n=237).

**Figure S3.**

**
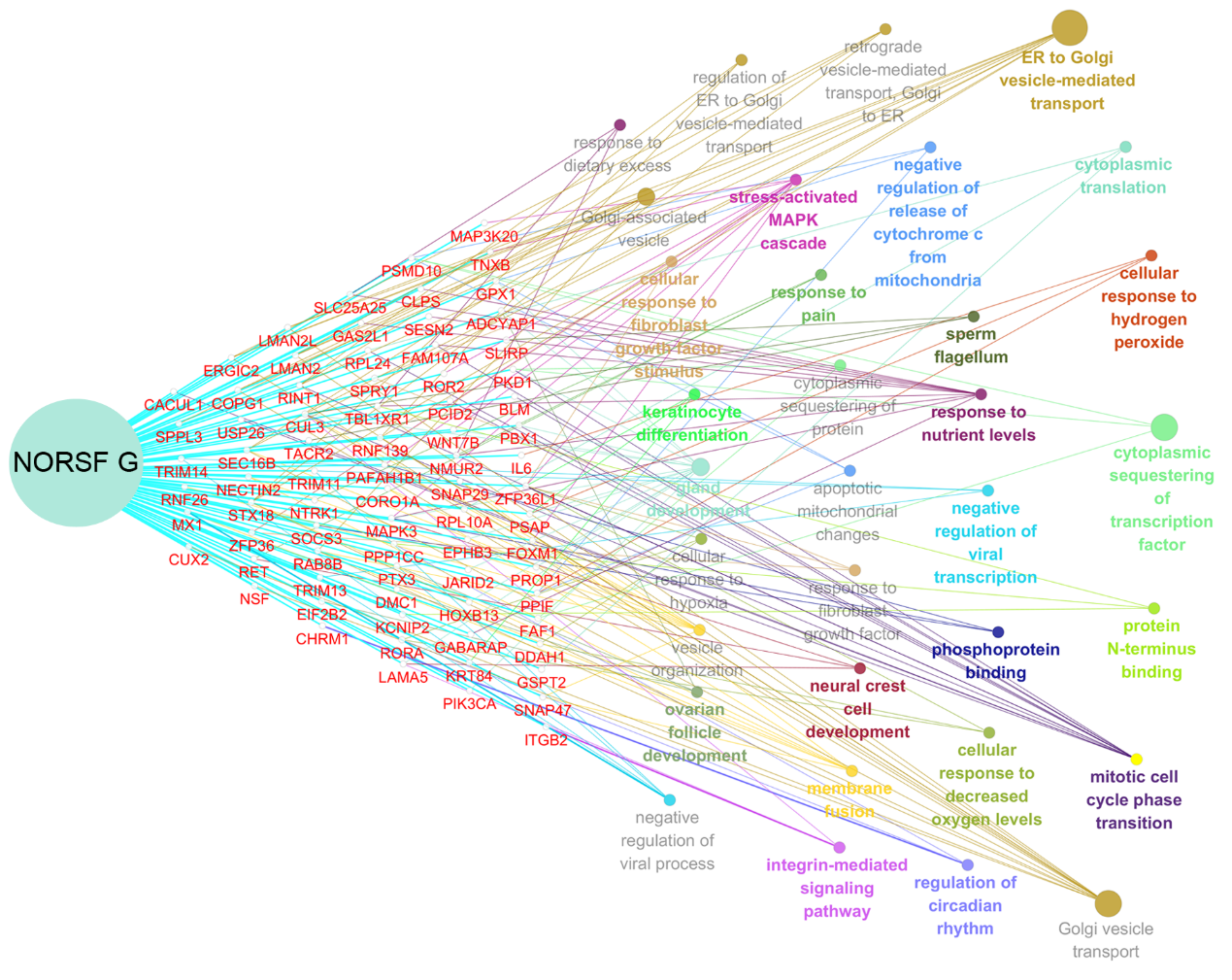
**

**Figure S3. Interaction network of RBPs interacting with *NORSF* transcript with Allele G.** Cytoscape v3.10.1 software was performed. GO terms are indicated by various node colour, and their size indicates the significance. The larger the node the more significant.

**Figure S4.**

**
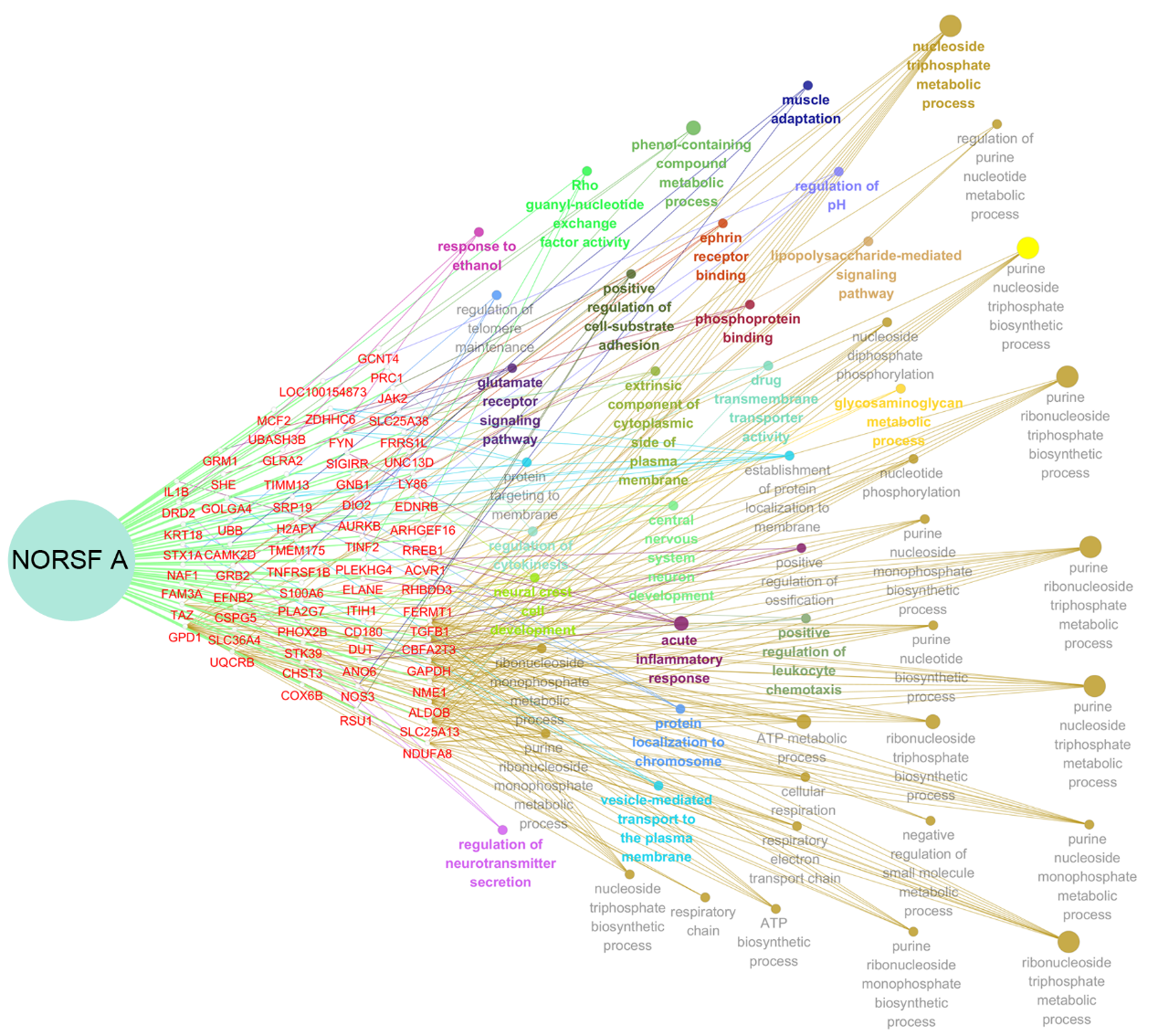
**

**Figure S4. Interaction network of RBPs interacting with *NORSF* transcript with Allele A.** Cytoscape v3.10.1 software was performed. GO terms are indicated by various node colour, and their size indicates the significance. The larger the node the more significant.

**Figure S5.**

**
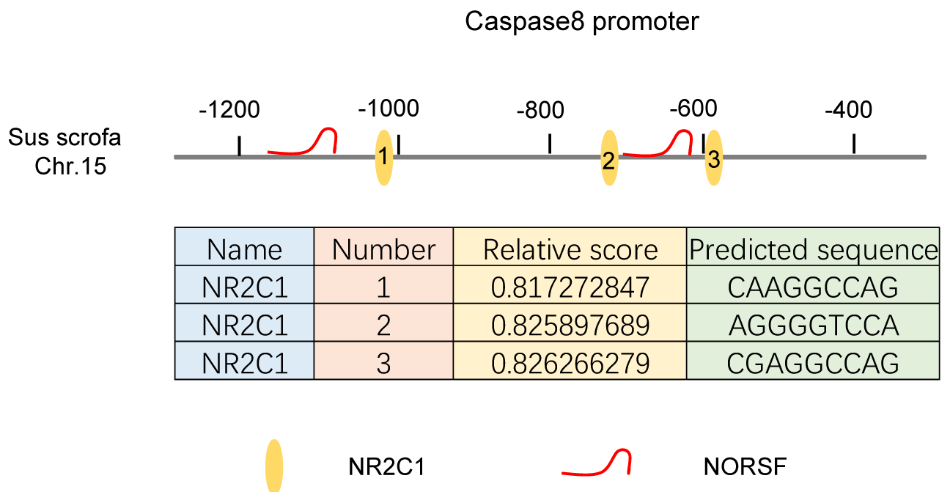
**

**Figure S5.** Schematic representation of the putative binding sites of *NORSF* and NR2C1 in the *Caspase8* promoter.

**Figure S6.**

**
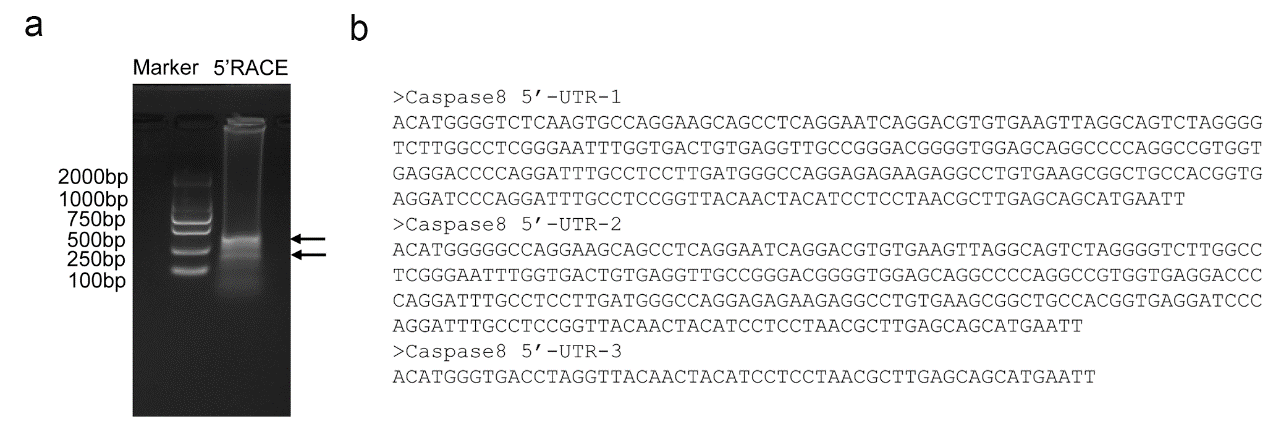
**

**Figure S6. Identification of the 5'-UTR of the pig *Caspase8* gene in sGCs.** (a) Gel images showing the products of 5′-RACE. Arrows indicate the target fragments. (b) The sequence of the 5'-UTR of the porcine Caspase8 gene.

**Figure S7.**

**
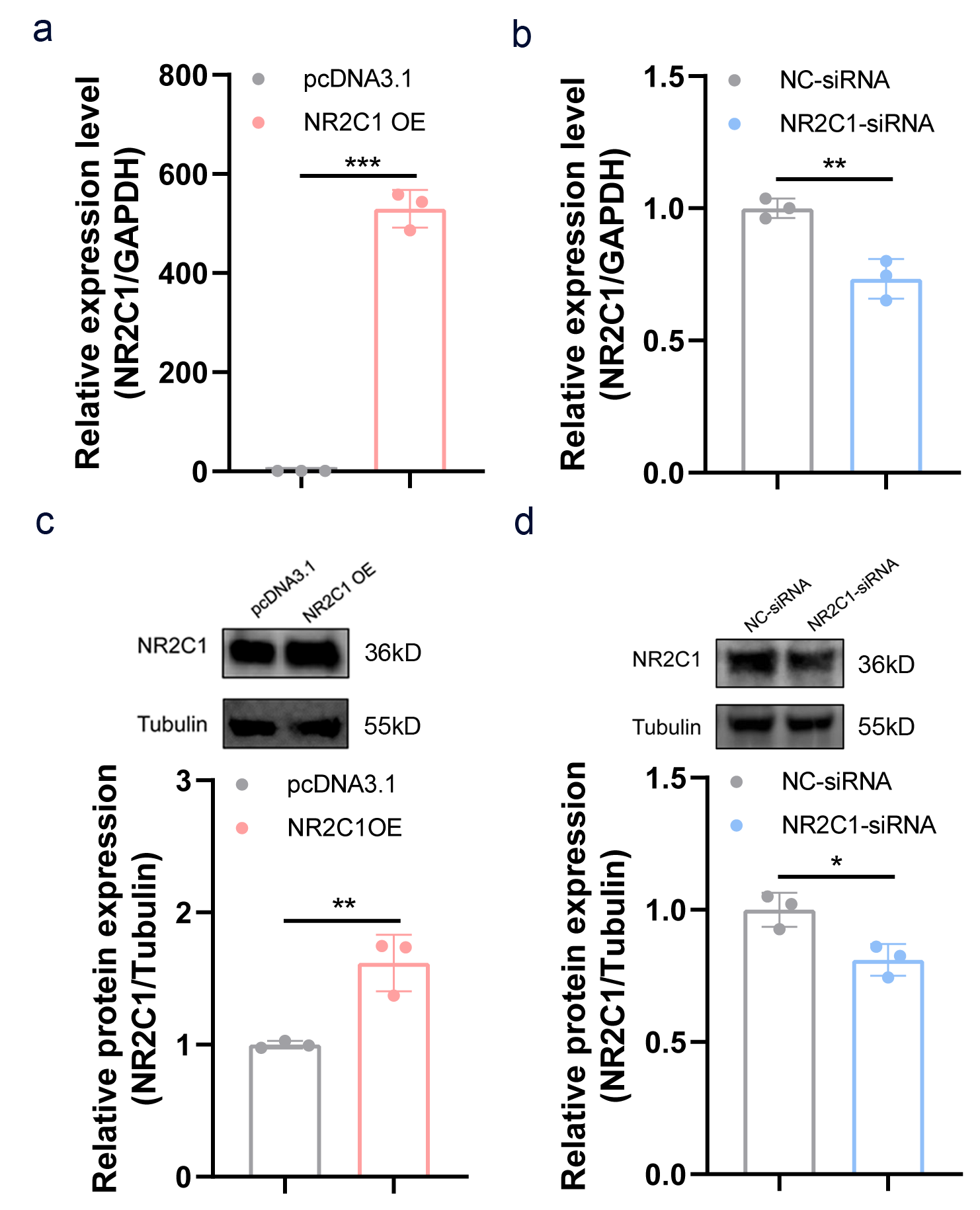
**

**Figure S7.** **Validation of overexpression and interference efficiency of NR2C1 in sGCs.** sGCs were transfected with pcDNA3.1-NR2C1 or NR2C1-siRNA, NR2C1 mRNA (a, b) and protein (c, d) levels were detected. n=3. Quantitative data are plotted as mean ± standard error. * P < 0.05. ** P < 0.01. *** P < 0.001.

**Figure S8.**


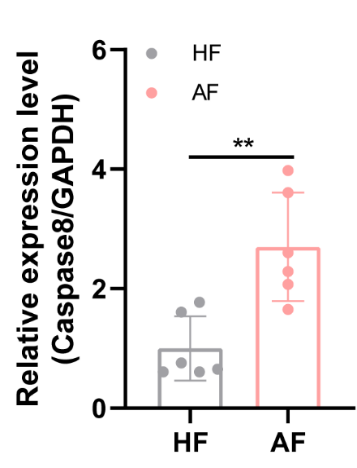


**Figure S8. *Caspase8* mRNA levels in sGCs from healthy follicles and atretic follicles.** HF, healthy follicle. AF, atretic follicle. n=6. Quantitative data are plotted as mean ± standard error. ** P < 0.01.

**Figure S9.**

**
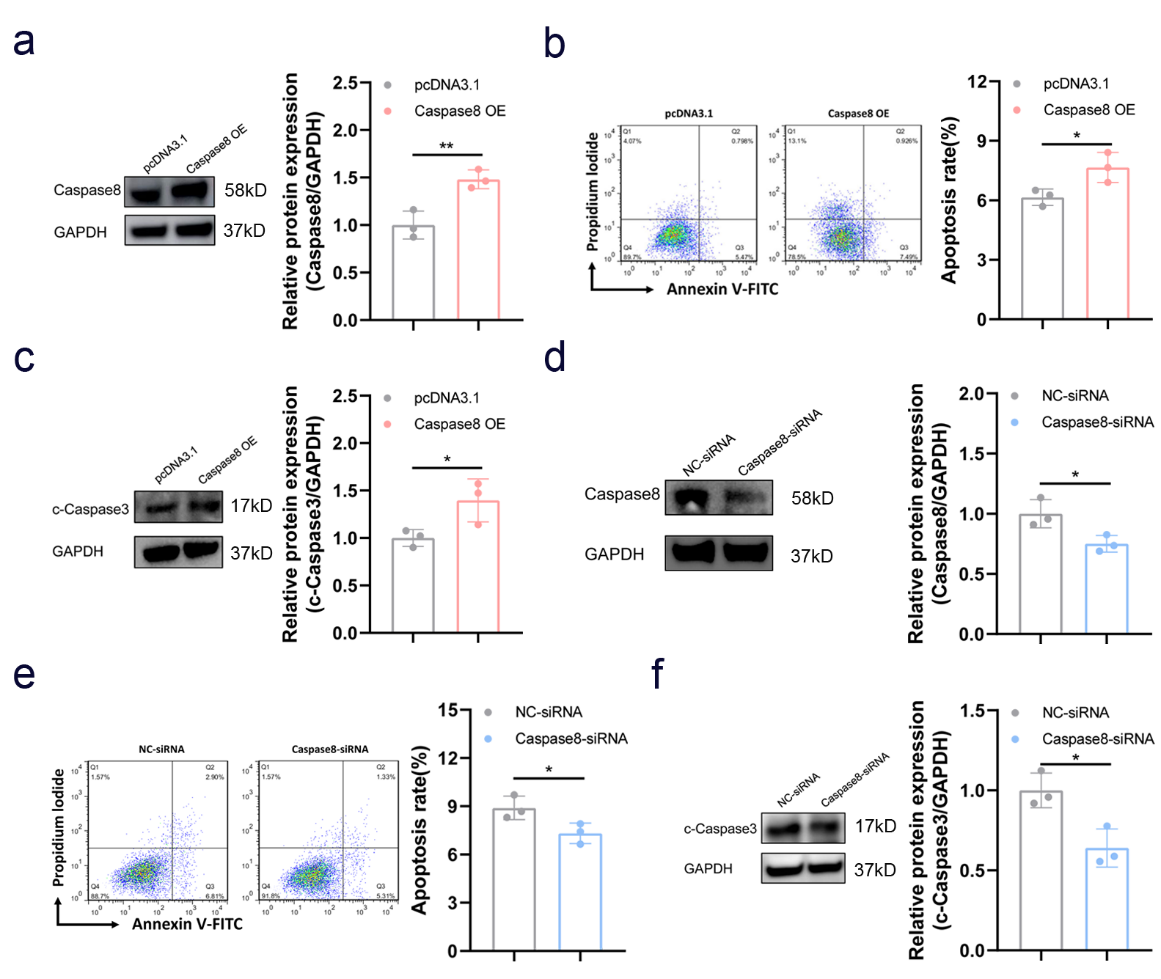
**

**Figure S9. Caspase8 is an apoptosis-promoting factors.** (a-c) sGCs were tansfected with pcDNA3.1-Caspase8, western blotting was conducted to detect Caspase8 (a) and c-Caspase3 (c) protein levels, and FACS was conducted to detect apoptosis rate (b). n=3. (d-f) sGCs were tansfected with Caspase8-siRNA, Caspase8 protein levels (d), apoptosis rate (e) and c-Caspase3 protein levels (f) were detected. n=3. Quantitative data are plotted as mean ± standard error. * P < 0.05. ** P < 0.01.

**Figure S10.**

**
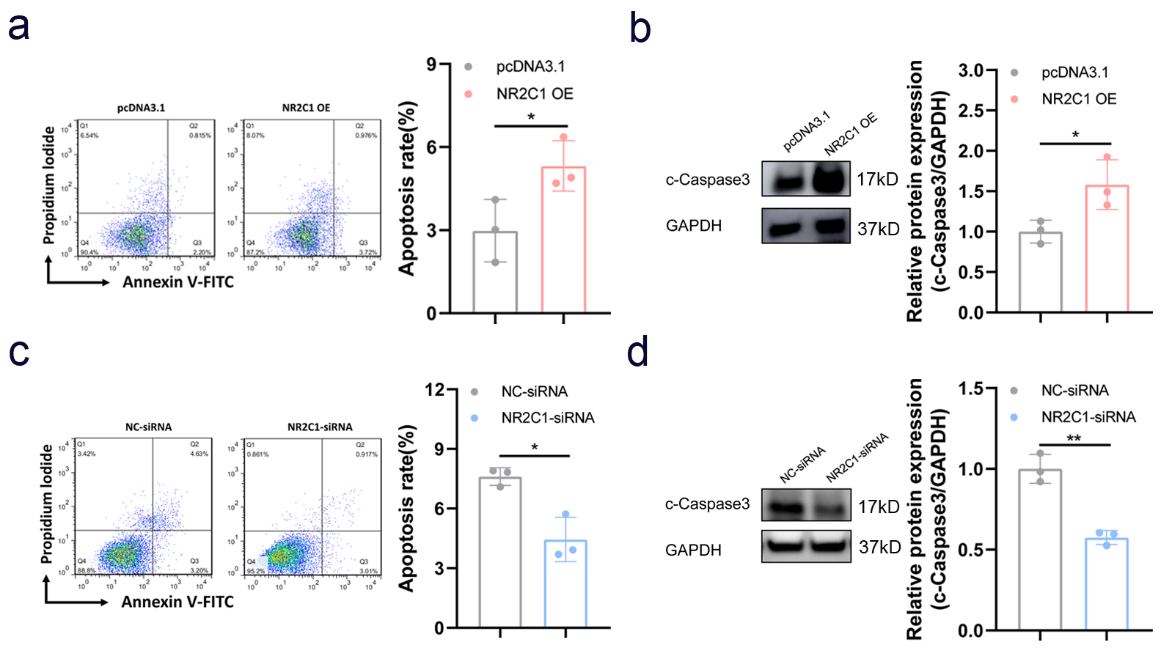
**

**Figure S10. NR2C1 is an apoptosis-promoting factor.** (a, b) sGCs were transfected with NR2C1 overexpression vector, and apoptosis rate was detected by FACS (a), c-Caspase3 protein levels were detected by western blotting (b). n=3. (c, d) sGCs were transfected with NR2C1-siRNA, and apoptosis rate (c) and c-Caspase3 protein levels (d) were detected. n=3. n=3. Quantitative data are plotted as mean ± standard error. * P < 0.05. ** P < 0.01.

**Figure S11.**

**
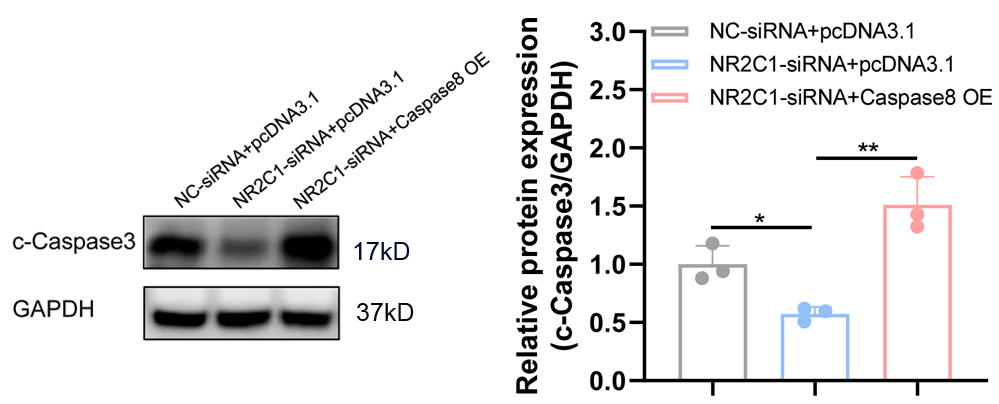
**

**Figure S11. NR2C1 activates the death receptor pathway in sGCs.** The c-Caspase3 protein levels in sGCs after co-transfection with NR2C1-siRNA and pcDNA3.1-Caspase8. n=3. Quantitative data are plotted as mean ± standard error. * P < 0.05. ** P < 0.01.

**Figure S12.**

**
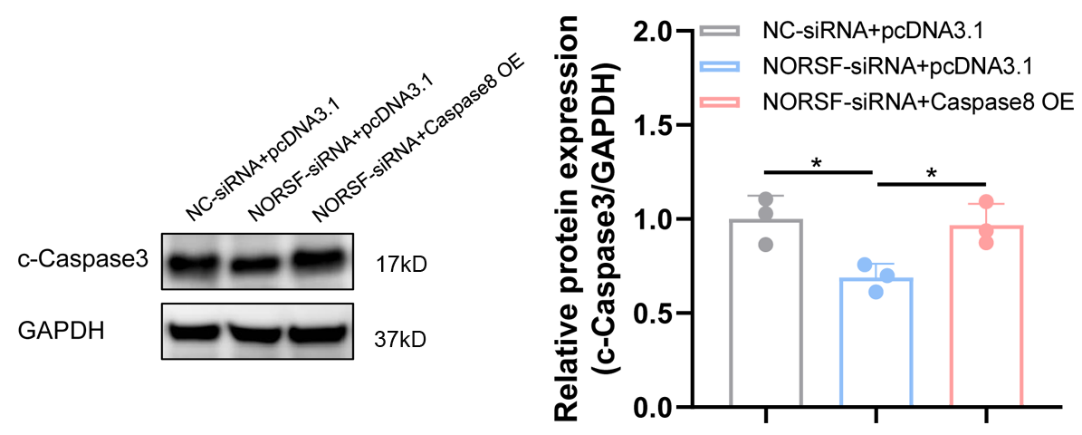
**

**Figure S12. *NORSF* regulates c-Caspase3 protein expression through Caspase8.** The c-Caspase3 protein levels in sGCs after co-transfection with NORSF-siRNA and pcDNA3.1-Caspase8. n=3. Quantitative data are plotted as mean ± standard error. * P < 0.05.

**Figure S13.**

**
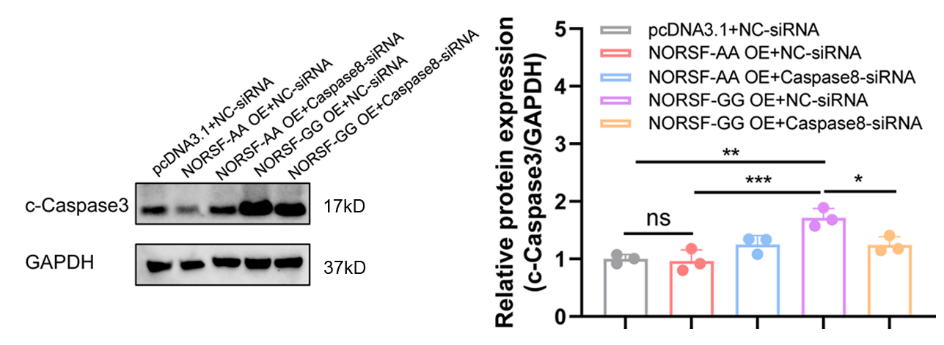
**

**Figure S13. *NORSF* g.128G>A mutation triggers sGC apoptosis through activating the death receptor pathway.** sGCs after co-transfection with *NORSF* transcript with genotype GG or genotype AA and Caspase8-siRNA, c-Caspase3 protein levels were detected using western blotting. n=3. Quantitative data are plotted as mean ± standard error. * P < 0.05. ** P < 0.01. *** P < 0.001. ns, not significant.
