# Supplementary material for: A Mutation Losing an RBP‐Binding Site in the LncRNA NORSF Transcript Influences Granulosa Cell Apoptosis and Sow Fertility
Source: Adv Sci (Weinh). 2024 Aug 9;11(40):2404747. doi: 10.1002/advs.202404747 (PMC11516108; doi:10.1002/advs.202404747)

ADVANCED SCIENCE
Open Access

Supporting Information

for *Adv. Sci.*, DOI 10.1002/advs.202404747

A Mutation Losing an RBP-Binding Site in the LncRNA NORSF Transcript Influences Granulosa Cell Apoptosis and Sow Fertility

*Miaomiao Wang, Wenmin Sheng, Jiyu Zhang, Qiuyu Cao, Xing Du and Qifa Li**

Figure 3f

pcDNA3.1	+	-	-	+	-	-	+	-	-
NORSF-AA OE	-	+	-	-	+	-	-	+	-
NORSF-GG OE	-	-	+	-	-	+	-	-	+

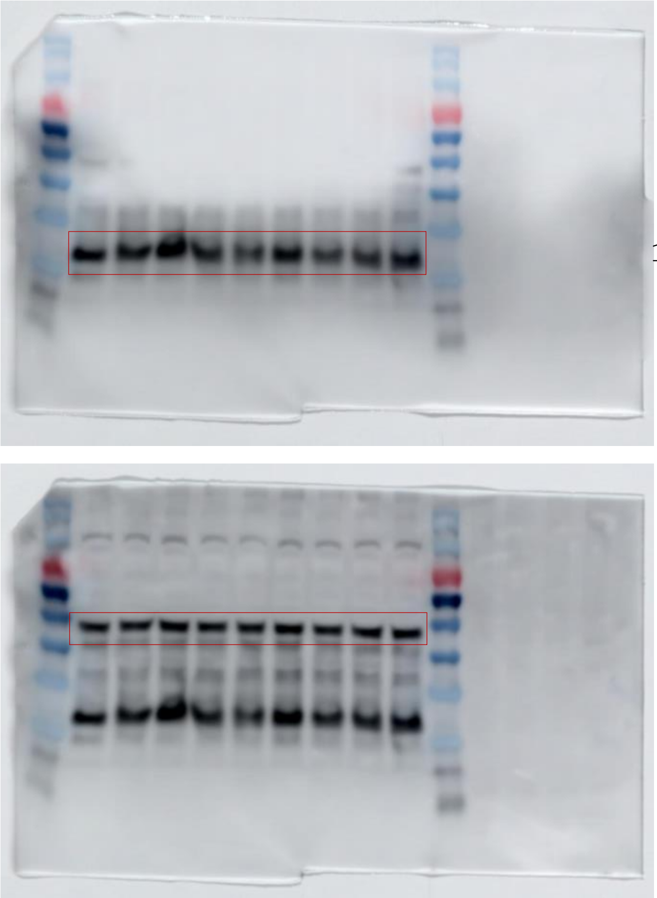

c-Caspase3 17kD

GAPDH 37kD

Figure 3i

NC-siRNA + − + − + −

NORSF-siRNA − + − + − +

c-Caspase3

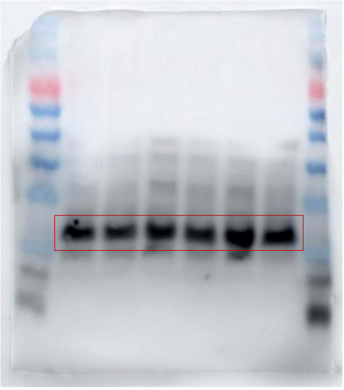

17kD

GAPDH

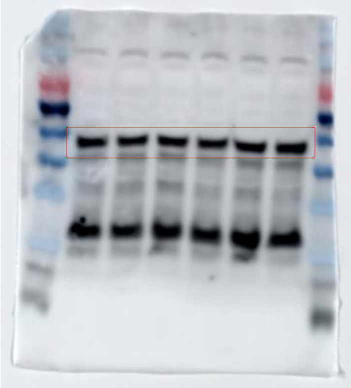

37kD

Figure 4b

RNA-pulldown input

NORSF A-Biotin	+	-	+	-	+	-
NORSF G-Biotin	-	+	-	+	-	+

NR2C1

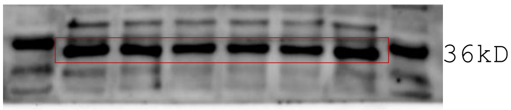

36kD

Tubulin

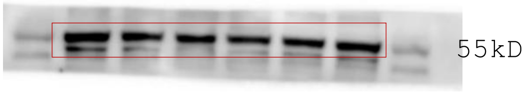

55kD

RNA-pulldown

NORSF A-Biotin	+	-	+	-	+	-
NORSF G-Biotin	-	+	-	+	-	+

NR2C1

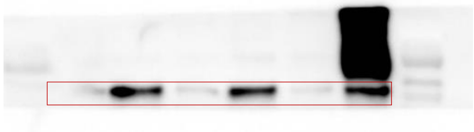

36kD

Figure 4h

pcDNA3.1	+	-	-	+	-	-	+	-	-
NORSF-AA OE	-	+	-	-	+	-	-	+	-
NORSF-GG OE	-	-	+	-	-	+	-	-	+

NR2C1

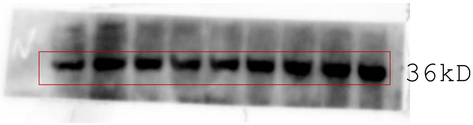

36kD

Tubulin

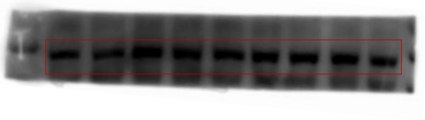

55kD

Figure 4j

NC-siRNA	+	-	+	-	+	-
NORSF-siRNA	-	+	-	+	-	+

NR2C1 — 36kD

Tubulin — 55kD

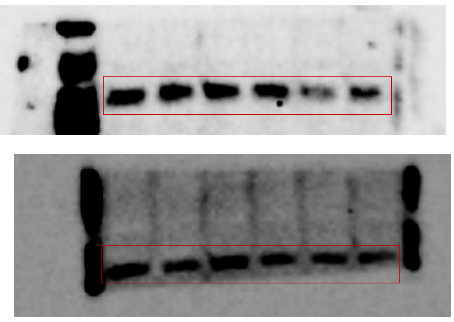

Figure 5h

pcDNA3.1	+	-	+	-	+	-
NR2C1 OE	-	+	-	+	-	+

Caspase8 — 58kD

GAPDH — 37kD

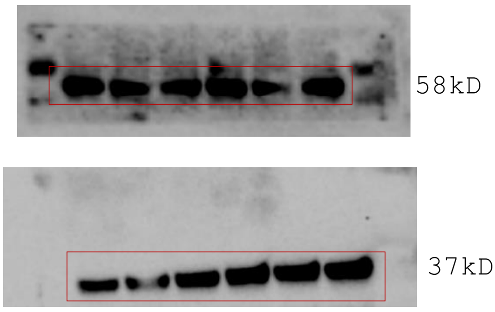

Figure 5i

NC-siRNA	+	-	+	-	+	-
NR2C1-siRNA	-	+	-	+	-	+

Caspase8 — 58kD

GAPDH — 37kD

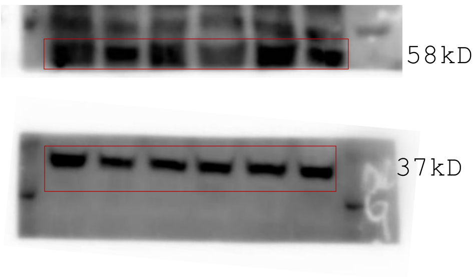

Figure 6b

pcDNA3.1 + - - + - - + - -

NORSF-AA OE - + - - + - - + -

NORSF-GG OE - - + - - + - - +

Caspase8

58kD

GAPDH

37kD

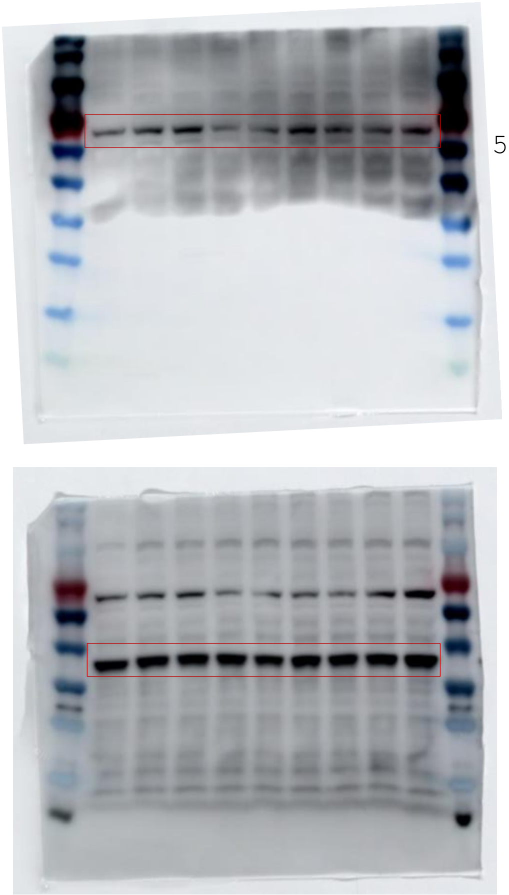

Figure 6d

NC-siRNA + - + - + -

NORSF-siRNA - + - + - +

Caspase8

58kD

GAPDH

37kD

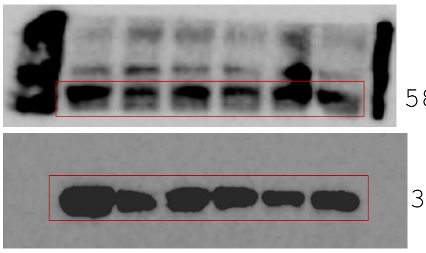

Figure S7c

pcDNA3.1 + - + - + -
NR2C1 OE - + - + - +

NR2C1 36kD

Tubulin 55kD

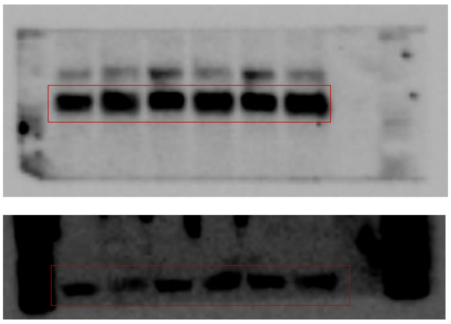

Figure S7d

NC-siRNA + - + - + -
NR2C1-siRNA - + - + - +

NR2C1 36kD

Tubulin 55kD

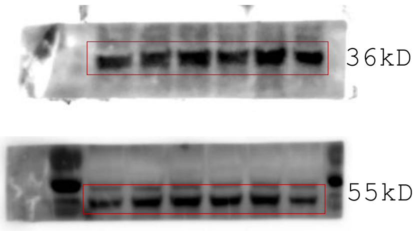

Figure S9a

pcDNA3.1 + − + − + −

Caspase8 OE − + − + − +

Caspase8 58kD

GAPDH 37kD

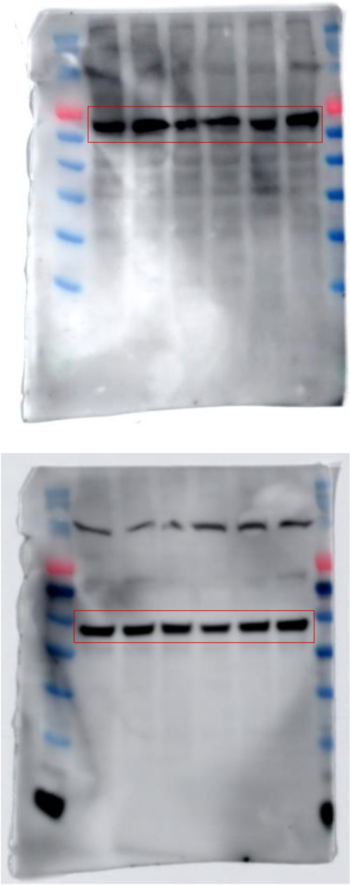

Figure S9c

pcDNA3.1 + − + − + −

Caspase8 OE − + − + − +

c-Caspase3 17kD

GAPDH 37kD

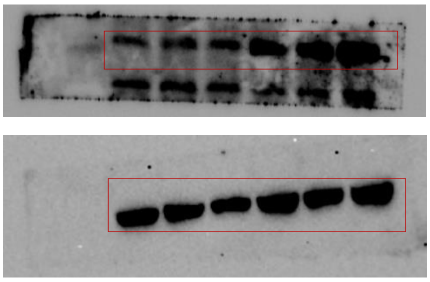

Figure S9d

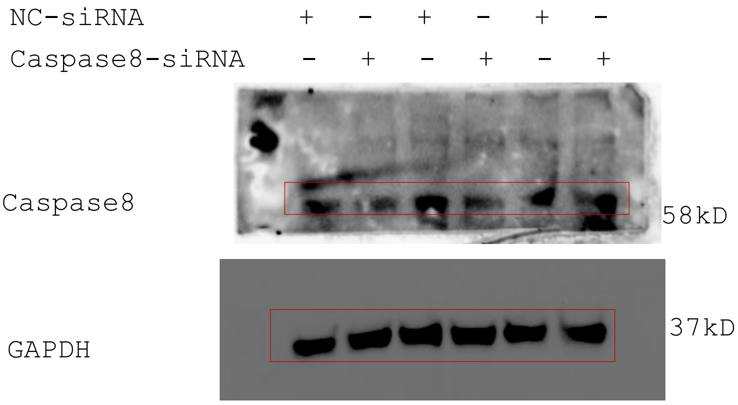

Figure S9f

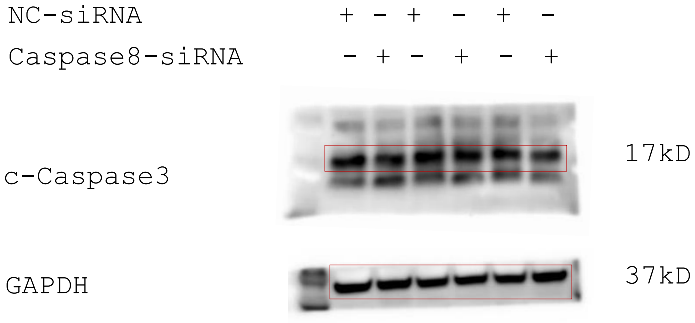

Figure S10b

pcDNA3.1	+	-	+	-	+	-
NR2C1 OE	-	+	-	+	-	+

c-Caspase3

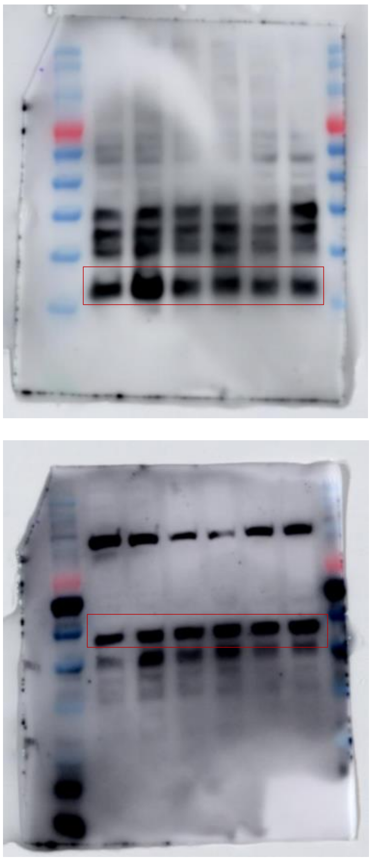

17kD

GAPDH

37kD

Figure S10d

NC-siRNA	+	-	+	-	+	-
NR2C1-siRNA	-	+	-	+	-	+

c-Caspase3

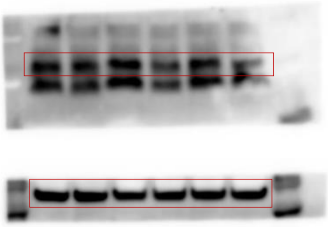

17kD

GAPDH

37kD

Figure S11

NC-siRNA + - - + - - + - -
NR2C1-siRNA - + - - + - - + -
Caspase8 OE - - + - - + - - +

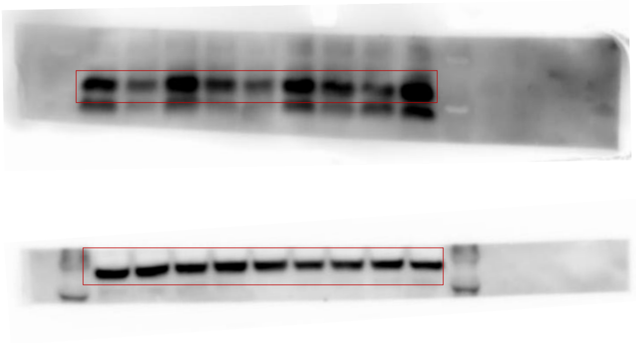

Figure S12

NC-siRNA + - - + - - + - -
NORSF-siRNA - + - - + - - + -
Caspase8 OE - - + - - + - - +

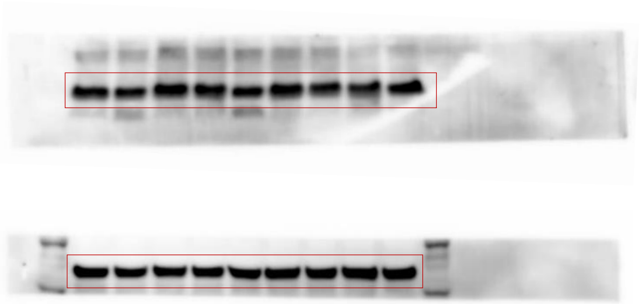

Figure S13

pcDNA3.1	+ - - - -	+ - - - - - + - - - -
NORSF-AA OE	- + - - -	- + - - - - + - - -
NORSF-GG OE	- - - + -	- - - + - - - - + -
NC-siRNA	+ + - + -	+ + - + - + + - + -
Caspase8-siRNA	- - + - +	- - + - + - - + - +

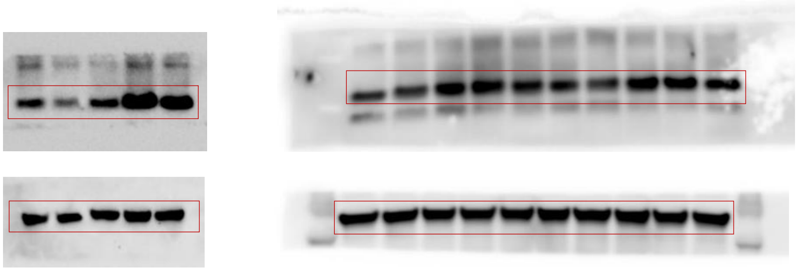

Supplement: Supplementary file 2 — Supporting Information [file ADVS-11-2404747-s002.pdf]
